# Ultrafast, High‐Capacity Uranium Harvesting From Seawater via a Hierarchically Porous Polymer Electrode

**DOI:** 10.1002/advs.75367

**Published:** 2026-04-20

**Authors:** Zejun Song, Zhenggong Wang, Lele Guo, Zhining Wang, Keming Wan, Ting Zhang, Wangxi Fang, Jian Jin

**Affiliations:** ^1^ School of Nano‐Tech and Nano‐Bionics University of Science and Technology of China Hefei China; ^2^ i‐Lab Suzhou Institute of Nano‐Tech and Nano‐Bionics Chinese Academy of Sciences Suzhou China; ^3^ State Key Laboratory of Bioinspired Interfacial Materials Science & College of Chemistry Chemical Engineering and Materials Science Soochow University Suzhou China; ^4^ Shandong Key Laboratory of Water Pollution Control and Resource Reuse School of Environmental Science and Engineering Shandong University Qingdao China

**Keywords:** electrochemical deposition, hierarchical porous electrode, polymer of intrinsic microporosity, solution‐processable polymer, uranium harvesting

## Abstract

Efficient uranium extraction from seawater is a critical challenge in advancing sustainable nuclear energy, given the vast but dilute reserves of uranium in oceans. Here, we report a hierarchically porous polymer electrode engineered for electrochemical uranium harvesting with unprecedented performance. Fabricated via a non‐solvent phase inversion process, the electrode features an interconnected microporous‐mesoporous architecture that enhances uranium uptake and facilitates ion transport, where the amidoxime groups promote selective adsorption of uranyl ions, and they are subsequently electrodeposited as crystalline (UO_2_)O_2_·2H_2_O under an applied potential. This solution‐processable microporous polymer electrode exhibits an excellent uranium uptake capacity exceeding 4221 mg g^−1^ without saturation. The electrode maintains strong reusability and seawater tolerance, enabling rapid and continuous uranium harvesting even at ultratrace concentrations. This work provides a transformative platform for electrochemically‐driven, high‐throughput uranium harvesting from marine resources.

## Graphical abstract

A hierarchically porous AOPIM‐CNT electrode enables uranium harvesting from seawater via adsorption‐promoted electrodeposition. Rapid diffusion and selective adsorption are coupled with electrochemical crystallization and spontaneous detachment, creating a self‐refreshing process that sustains high‐capacity, continuous extraction from ultradilute marine environments.

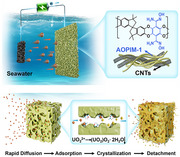

## 1 Introduction

Nuclear energy, with its high energy density and low carbon emissions, plays a critical role in the global transition toward sustainable energy [1]. While current nuclear power generation relies almost entirely on terrestrial uranium resources, these reserves are projected to be depleted within this century. In contrast, the ocean harbors over 4.5 billion metric tons of uranium—roughly 1000 times more than land‐based deposits—offering a virtually inexhaustible supply of nuclear fuel [2, 3]. However, the ultralow concentration of uranium in seawater (∼3.3 µg L^−1^) and the presence of competing ions pose significant scientific and technological challenges for extraction [4, 5, 6, 7, 8, 9, 10, 11, 12, 13]. Recognizing its strategic importance, uranium extraction from seawater (UES) has been identified as one of the “Seven Chemical Separations to Change the World” [14].

UES technologies have progressed through three key phases: (1) early‐stage adsorption materials [15], (2) functional polymer adsorbents [16, 17, 18, 19], and (3) emerging electro‐assisted systems [20, 21, 22, 23, 24, 25, 26]. Among them, amidoxime‐functionalized polymers have dominated due to their strong affinity for uranyl ions [27, 28]. Yet, conventional adsorbents remain limited by sluggish adsorption kinetics and poor selectivity, typically achieving capacities below 7 mg g^−1^ [29]. More recently, external fields‐driven approaches [30, 31], such as photo [32] and alternating current electrodeposition [25, 33], have shown promise in overcoming these barriers by directly reducing uranyl ions under applied voltages. In particular, half‐wave rectified alternating current electrochemistry (HW‐ACE) enables dynamic control over ion selectivity via electrical double‐layer regulation and facilitates the electrochemical conversion of U(VI) into insoluble uranium peroxide precipitates. This strategy breaks both the kinetic and thermodynamic constraints of physisorption, allowing continuous uranium harvesting. Subsequent advancements in electrode materials include Yang et al.’s iron single‐atom decorated hollow nitrogen‐doped carbon capsules with amine‐oxime functionalization, achieving redox conversion of U(VI) into Na_2_O(UO_3_·H_2_O)_x_ precipitates, and reducing seawater uranium levels from 3.5 ppb to below 0.5 ppb within 24 h with 1.2 mg g^−1^ extraction capacity [34]. Similarly, Wang et al. reported a hydrogen‐bonded melamine‐phenanthroline porous framework (MPSOF) that effectively converts uranyl to uranium peroxide precipitates [35]. However, these powder‐based systems suffer from binder‐related degradation and inadequate mechanical integrity, and their architecture offers limited control over uranyl ion diffusion and adsorption efficiency.

**FIGURE 1:**
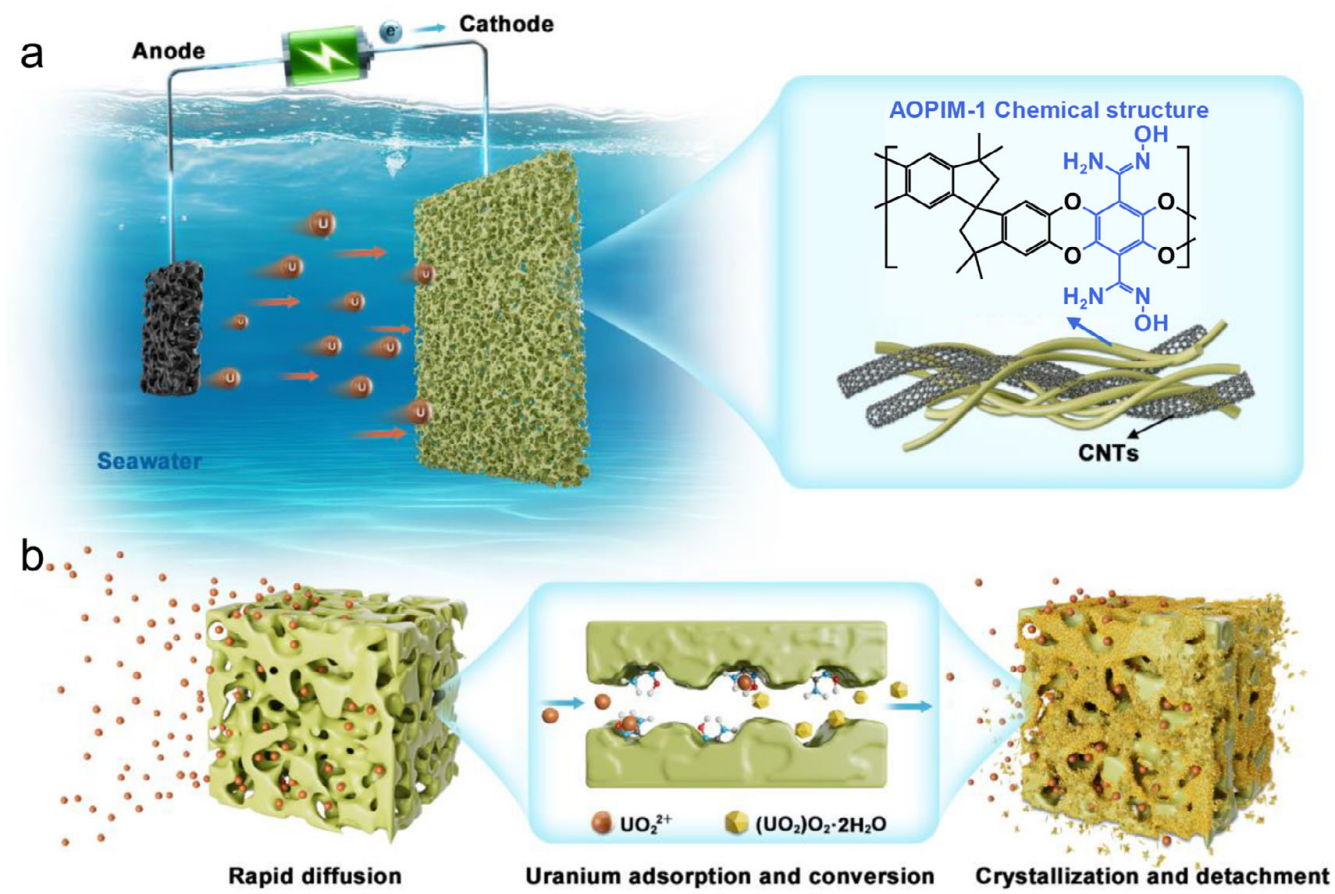
Schematic illustration of the AOPIM‐CNT composite electrode and electrochemical uranium extraction. (a) Overview of the AOPIM‐CNT electrode enabled seawater uranium extraction under half‐wave rectified alternating current electrochemistry (HW‐ACE) conditions, chemical structure of the amidoxime‐functionalized PIM‐1 (AOPIM‐1) polymer and schematic representation its composite integration with hydroxylated carbon nanotubes (CNTs); (b) Mechanistic diagram illustrating uranyl ion diffusion, adsorption, electrochemical reduction, crystallization as (UO_2_)O_2_·2H_2_O, and detachment from the polymer matrix.

Polymers of intrinsic microporosity (PIMs) emerge as a promising alternative. Their molecular architecture, characterized by twisted rotatable bonds interconnected with rigid segments, inherently inhibits effective chain stacking and generates a network of intrinsic micropores [36, 37]. This unique structural configuration confers ultrahigh specific surface area, interconnected porous networks for rapid ion diffusion, and extensive exposure of active sites for uranyl binding [38]. Importantly, their solubility in common organic solvents makes them easily processable into functional electrodes. In this study, we present a hierarchically porous composite electrode composed of amidoxime‐functionalized PIM‐1 (AOPIM‐1) and hydroxylated carbon nanotubes (CNTs) for ultrafast, high‐capacity electrochemical uranium extraction (Figure 1). The amidoxime groups inside micropores of AOPIM‐1 provide abundant adsorption sites for uranium adsorption, while CNTs strengthen electrical conductivity and promote composite uniformity through hydrogen bonding interactions. Using a non‐solvent (ethanol)‐induced phase inversion technique, we fabricated electrodes with a unique sponge‐like porous architecture that facilitates rapid ion diffusion and maximize adsorption sites exposure. Under HW‐ACE conditions, the AOPIM‐CNT electrode exhibits an excellent uranium uptake of 4221 mg g^−1^ in a single cycle, without signs of saturation. Moreover, the system maintains robust performance across multiple extraction‐elution cycles in real seawater, extracting 2.19 µg U per liter of natural seawater in 24 h. This study introduces a structurally engineered polymer‐based electrode platform for scalable and sustainable uranium recovery from seawater.

## 2 Results and Discussion

### 2.1 Preparation and Characterization of Electrodes

Polymer of intrinsic microporosity (PIM‐1) was synthesized via polycondensation of 5,5′,6,6′‐tetrahydroxy‐3,3,3′,3′‐tetramethylspirobisindane (TTSBI) and tetra‐fluoroterephthalonitrile (TFTPN) (Figure S1) and subsequently functionalized with hydroxylamine to yield AOPIM‐1 (Figure S2) [36]. The chemical structure of AOPIM‐1 was confirmed by ^1^H nuclear magnetic resonance in Figure S3, while pore size distribution measurements (Figure S4) validated its intrinsic microporous structure. Conventional electrode fabrication typically involves slurry casting of granular active materials with polymeric binders to ensure mechanical integrity, followed by solvent evaporation to achieve component homogeneity [39, 40, 41]. However, this approach often leads to partial pore collapse during solvent removal, and the polymeric binders inevitably create interfacial barriers between active materials and reactants. In contrast, our AOPIM‐CNT electrode employs AOPIM‐1 as both the active material and structural binder, incorporating with an appropriate amount of hydroxylated carbon nanotubes to improve conductivity. This innovative configuration significantly increases the active material mass fraction while eliminating interfacial impedance from conventional binders. Owing to the exceptional solvent processability of AOPIM‐1, binder‐free electrodes with more tunable porosity can be fabricated through controlled phase inversion techniques.

**FIGURE 2:**
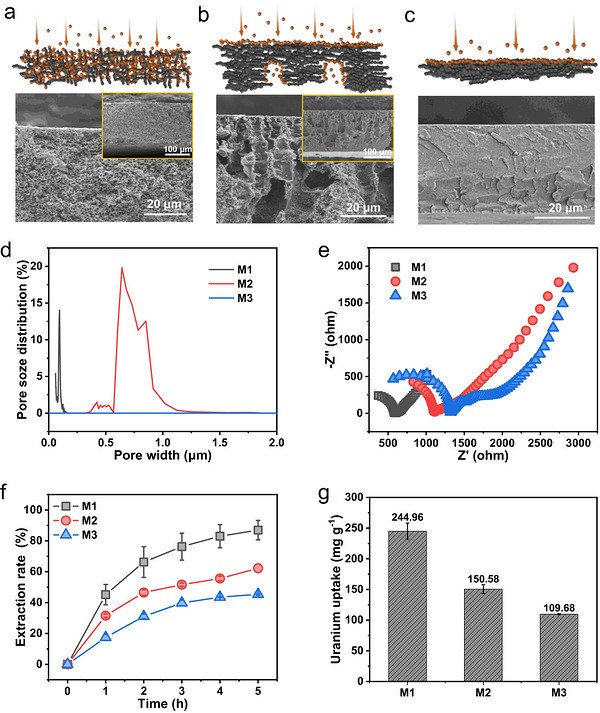
Influence of electrode fabrication methods on morphology, ion transport, and uranium extraction performance. SEM images and corresponding schematics showing ion diffusion behavior in electrodes fabricated by (a) ethanol‐induced phase inversion (M1), (b) water‐induced phase inversion (M2), and (c) solvent evaporation (M3); (d) Pore size distributions, (e) electrochemical impedance spectra in 50 ppm uranyl nitrate solution, (f) time‐dependent uranium extraction rates, and (g) uranium extraction capacities within 5 h for electrodes fabricated by the three different methods.

To compare fabrication methods, electrodes were prepared by ethanol‐induced phase inversion (M1), water‐induced phase inversion (M2), and solvent evaporation (M3), as illustrated in Figure S5. SEM images (Figure 2a–c) show that M1 forms delicate sponge‐like mesoporous networks, whereas M2 generates dense epidermal layers and finger‐shaped macropores, and M3 results in a dense, nonporous structure. Corresponding pore size distributions (Figure 2d) confirm ∼100 nm pores for M1, ∼700 nm for M2, and negligible porosity for M3. Water uptake tests (Figure S6) further confirmed the high porosity of M1 (64.2%) and M2 (70.0%) compared with M3 (29.8%). Based on the pore size distribution, M1 has a larger number of pores. The EIS data (Figure 2e) were fitted using an equivalent circuit model (Figure S7) to analyze the charge transfer and diffusion behaviors of electrodes with different pore structures. The results demonstrate that M1 exhibits the lowest charge transfer resistance (500 Ω) and Warburg impedance (404 Ω), significantly outperforming the finger‐pore electrode (*R*_ct_ ≈ 860 Ω, *W*_s_ ≈ 586 Ω) and the pore‐free electrode (*R*_ct_ ≈ 1000 Ω, *W*_s_ ≈ 580 Ω). This indicates that the sponge‐like hierarchical pore structure synergistically optimizes both charge transfer and mass diffusion. Specifically, it enhances charge transfer by increasing the electrochemically active area, while simultaneously reducing diffusion impedance by disturbing and thinning the diffusion layer. Furthermore, the similar Warburg impedances observed for the finger‐pore and pore‐free electrodes suggest that in this testing system, the resistance of the external diffusion layer predominantly governs the overall Warburg impedance. AOPIM‐CNT electrodes fabricated via different methods were evaluated as cathodes, paired with activated carbon anodes for uranium extraction (Figure S8). Optical photographs confirm the distinct morphologies of the AOPIM‐CNT (gray–yellow) and activated carbon (black) electrodes (Figure S9). The electrochemical extraction was carried out in a custom‐designed capacitive deionization apparatus (Figure S10).

A half‐wave rectified alternating current electrochemistry (HW‐ACE) strategy, as illustrated in Figure S11, was adopted to facilitate uranium extraction by applying square wave voltages at specific frequencies to drive ion migration. By optimizing the voltage parameters, the migration behavior was significantly improved. Experimental results indicated a strong positive correlation between the square wave voltage amplitude and the uranium extraction rate (Figure S12). Detailed analysis of the amperometric *i*–*t* curves (Figure S13) showed that setting the square wave voltage to 0–5 V with a frequency of 400 Hz led the system into a non‐continuous conductive state, effectively reducing the water electrolysis reaction. Compared to static adsorption and direct current (DC) modes, the HW‐ACE mode exhibited superior uranium extraction performance while minimizing water electrolysis (Figure S14). Consequently, based on these observations, an AC potential ranging from ‐5 to 0 V at 400 Hz was identified as the optimal operating condition.

The uranium concentration was monitored by UV–vis absorption at 652 nm after complexation with Arsenazo(III), calibrated against a standard curve (Figure S15) [38]. Strikingly, the sponge‐pore electrodes delivered the most efficient uranium extraction, removing ∼90% of uranium from within 5 h and achieving capacities up to 244.96 mg g^−1^ (Figure 2f,g). These results underscore the importance of structural engineering, where interconnected meso‐ and micropores facilitate rapid ion transport and adsorption, synergizing with HW‐ACE to accelerate electrochemical uranium deposition.

### 2.2 Influence of Electrode Composition

**FIGURE 3:**
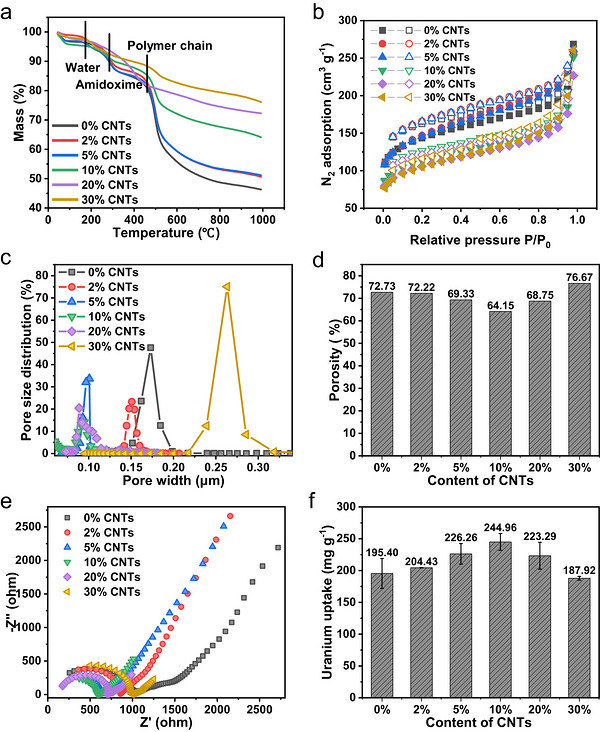
Effect of CNTs content on the structure and performance of AOPIM‐CNT composite electrodes. (a) Thermogravimetric analysis (TGA), (b) N_2_ adsorption–desorption isotherms, (c) pore size distributions, (d) porosity obtained from water uptake measurements, (e) electrochemical impedance spectra in 50 ppm uranyl nitrate solution, and (f) 5 h uranium extraction capacities of AOPIM‐CNT composite electrodes with varying CNTs content.

Electrodes with carbon nanotube contents of 0~30% were prepared by the ethanol‐induced phase inversion method to further investigate the optimal ratio of AOPIM and CNTs. FTIR (Figure S16) and TGA (Figure 3a) confirmed successful composite electrode preparation. SEM images (Figure S17) showed consistent sponge‐like morphologies with enlarging pores, while excess CNTs loading (> 40%) disrupted electrode integrity (Figure S18).

Brunauer–Emmett–Teller (BET) analysis (Figure 3b; Figure S19) shows that the pristine AOPIM‐1 has a specific surface area of 526 m^2^ g^−1^. Doping with 2∼5% CNTs causes negligible changes to the surface area, but increasing the loading to 10% reduces it to 415 m^2^ g^−1^. This reduction is attributed to interfacial interactions between hydroxyl‐functionalized CNTs and AOPIM‐1 chains, which restrict chain mobility and lead to denser chain packing, thereby diminishing the intrinsic microporous volume. The macropore size exhibits a non‐monotonic trend with varying CNTs content (Figure 3c,d). At low CNTs loadings (< 10%), the macropore size decreases from approximately 170 to 100 nm, accompanied by a drop in water uptake from 72.73% to 64.15%, as the incorporation of CNTs increases the viscosity of the electrode slurry, hindering phase separation. Conversely, at high CNTs loadings (> 20%), the macropore size rapidly increases to approximately 250 nm, and water uptake rises to 76.67%, due to the agglomeration of CNTs at higher contents, which introduces defects and compromises structural integrity. Consequently, the optimal performance at 10% CNTs represents a balanced trade‐off between enhanced conductivity and the preservation of pore structure.

To analyze the impact of CNTs loading on charge transfer and diffusion behaviors, we utilized an equivalent circuit model (Figure S7) to fit the EIS data of electrodes with different CNTs contents (Figure 3e). As the CNTs content increases, *R*_ct_ decreases from 796 Ω at 0% to a minimum of 507 Ω at 10%, then rises to 848 Ω at 30%. This indicates that an appropriate amount of CNTs (5~10%) can form a conductive network and optimize the pore structure, thereby synergistically reducing charge transfer resistance and diffusion impedance. However, when the CNTs content reaches or exceeds 20%, CNTs agglomeration disrupts the conductive network, while the reduced content of the active component AOPIM‐1 leads to fewer effective adsorption sites, making it difficult for uranyl ions to bind effectively even after diffusing into the electrode, resulting in a significant increase in *R*_ct_. In contrast, *W*_s_ rapidly decreases from 763 Ω at 0% to approximately 400 Ω in the 5~10% range, and further declines to 155 Ω at 30%. This decreasing trend suggests that structural defects within the electrode provide larger diffusion channels for ions, but this usually comes at the expense of structural integrity and does not translate into performance advantages. Based on the combined trends of *R*_ct_ and *W*_s_, 10% CNTs is identified as the optimal ratio, where charge transfer is most favorable, diffusion performance is good, and the electrode structure remains intact. Further uranium extraction experiments (Figure 3f) confirmed that the electrode with 10% CNTs delivered the highest uranium extraction efficiency. Performance is dictated by the combined effects of ion diffusion/adsorption and electrode conductivity, with this composition providing the optimal synergy. Under these conditions, the AOPIM‐CNT electrode successfully achieved rapid, adsorption‐promoted electrodeposition of uranyl ions, fully consistent with the mechanistic trends described above. Consequently, we proceeded with further experiments using this optimized electrode configuration.

### 2.3 Uranium Extraction From Freshwater

**FIGURE 4:**
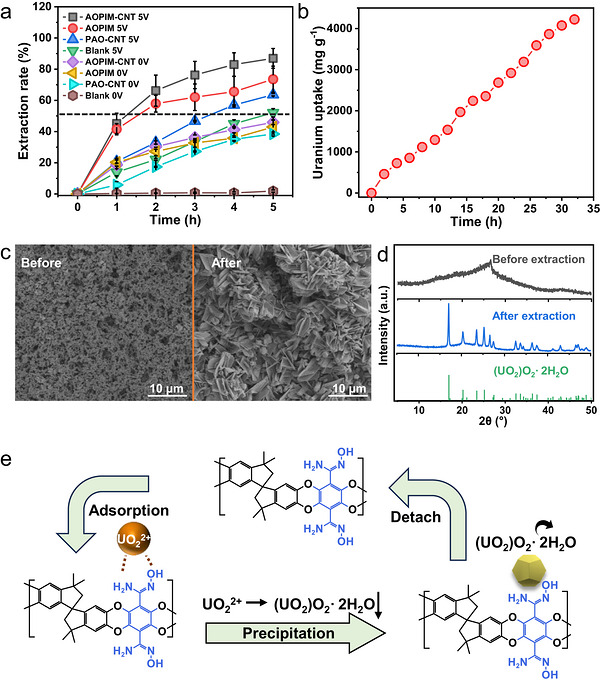
Characterization of uranium electrodeposition behavior. (a) Uranium extraction rate over time under different voltages and electrode types; (b) Uranium extraction under large‐scale (bulk) operation; (c) SEM image of the electrode surface before and after uranium deposition; (d) XRD spectra of pristine AOPIM‐CNT, electrode after deposition, and simulated (UO_2_)O_2_·2H_2_O; (e) Schematic mechanism of AOPIM‐1 enabled uranyl adsorption, electrochemical uranium reduction and precipitation, (UO_2_)O_2_·2H_2_O detachment and electrode regeneration.

The uranium extraction performance of AOPIM‐CNT electrodes was benchmarked against AOPIM‐1, Polyacrylamide oxime (PAO)‐CNT (Figure S21), and blank carbon paper controls. Without external voltage, both AOPIM‐1 and AOPIM‐CNT electrodes exhibit excellent static adsorption performance by removing ∼43% uranium ions from 50 mL of 50 ppm uranyl solution within 5 h, outperforming PAO‐CNT (∼38%) and blank carbon paper electrodes (∼0%) as shown in Figure 4a. This confirms the role of intrinsic microporosity in enhancing adsorption. Under HW‐ACE, the AOPIM‐CNT electrode extracted 86.9% of uranium within 5 h, far exceeding PAO‐CNT (63.6%) and AOPIM‐1 (73.5%), highlighting the synergistic effects of conductivity and microporosity. Specifically, the intricate microporous architecture of the AOPIM‐CNT electrodes facilitates rapid ion diffusion and adsorption, accelerating electrochemical reactions and promoting efficient uranium deposition. Interestingly, even the blank electrode can extract ∼52% of uranium under HW‐ACE, demonstrating the general effectiveness of this technique. Continuous uranium extraction experiments (Figure 4b) revealed a nearly constant uranium uptake rate of AOPIM‐CNT electrodes, achieving an excellent capacity of 4221 mg g^−1^ in a single cycle without saturation. SEM imaging (Figure 4c) and EDS mapping (Figures S23 and S24) confirmed significant uranium crystal deposition on the electrode surface.

The effect of pH on the uranium extraction performance of the AOPIM‐CNT electrode was further investigated. Zeta potential measurements (Figure S25a) showed that the electrode surface charge shifted from positive under acidic conditions (pH < 5) to negative at neutral and alkaline conditions, consistent with amidoxime protonation–deprotonation behavior (Figure S25b). Visual MINTEQ simulations (Figure S26) indicated a complementary trend for uranyl speciation, where uranyl ions are positively charged under acidic conditions, and negatively charged under alkaline conditions [9, 42]. Optimal performance occurred at pH 7~8, where negatively charged AOPIM‐1 favorably interacted with positively charged uranyl species (Figure S27). This coincides with the natural seawater pH (∼8), underscoring the electrode's suitability for practical uranium harvesting [38]. Interestingly, uranyl ions in seawater do not follow typical hydrolysis behavior but instead exist predominantly as electrically neutral or anionic tricarbonato complexes, [UO_2_(CO_3_)_3_] [4−]. Despite the thermodynamic stability of these carbonate complexes, amidoxime groups exhibit a remarkable ability to displace carbonate ligands and coordinate directly with uranyl ions, demonstrating exceptional binding affinity even under seawater conditions [9].

The ion‐selective behavior of the electrode was investigated at pH 8.0 (Figure S28). After operating for 12 h under an applied electric field, the uranium extraction efficiency was significantly higher than that of vanadium and iron, which also exhibit strong complexation with amidoxime groups. This signifies the practicality of the present strategy with respect to uranium ion selectivity.

### 2.4 Uranium Electrodeposition Mechanism

Electrochemical reactions at the AOPIM‐CNT electrode during uranium extraction were investigated by cyclic voltammetry (CV) in various background solutions, with and without uranyl ions. As depicted in Figure S29a, in a NaCl background solution, the CV curves show a reduction peak corresponding to dissolved oxygen at approximately −0.3 V both in the presence and absence of uranyl ions. Differently, upon the addition of uranyl ions, a new reduction peak appears near ‐1 V and a new oxidation peak appears near ‐0.4 V. These might correspond to the reduction of U(VI) to U(V) and the oxidation of U(V) to U(VI), respectively. Figure S29b displays the CV curves recorded in seawater background. While the general trend remains consistent with that observed in NaCl background solution, the oxidation and reduction potentials of each peak are shifted to lower values, which can be attributed to the higher concentration of seawater. Testing of DC mode (Figure S29c) further revealed that increasing the DC voltage from 0 to 0.8 V resulted in little improvement in uranium extraction performance, while a significant improvement was observed when the voltage was increased to 1 V, signifying the occurrence of electrochemical uranium deposition at ∼1 V. Structural analyses of the deposits by XRD and Raman spectroscopy revealed that the product was not uranyl nitrate from the solution but uranium peroxide, (UO_2_)O_2_·2H_2_O (Figure 4d; Figure S30a–c), consistent with literature reports [33, 35, 43]. Quasi‐in situ Raman spectroscopy indicates that uranium precipitation only occurs upon increasing the voltage to 1 V (Figure S30d). Furthermore, XPS (Figure S30e,f) analysis of this precipitate confirms the uranium species as U(VI). This pathway involves the electrochemical reduction of U(VI) to U(V) (Equation 1), followed by rapid reaction with dissolved oxygen to form insoluble uranium peroxide (Equation 2).

(1) $$UO_{2}^{2+}+e^{-}\to UO_{2}^{+}$$

(2) $$4UO_{2}^{+}+3O_{2}+10H_{2}O\to 4\left(UO_{2}\right)O_{2}\cdot 2H_{2}O↓+4H^{+}$$

Together with adsorption measurements, these results suggest a self‐refreshing process (Figure 4e): uranyl ions are rapidly diffused into the electrode and adsorbed onto amidoxime, electrochemically reduced to U(V), and converted into uranium peroxide precipitates. These precipitates then detach from the active sites, continuously regenerating adsorption sites and enabling rapid, sustained uranium extraction [44, 45].

### 2.5 Uranium Extraction From Spiked and Natural Seawater

**FIGURE 5:**
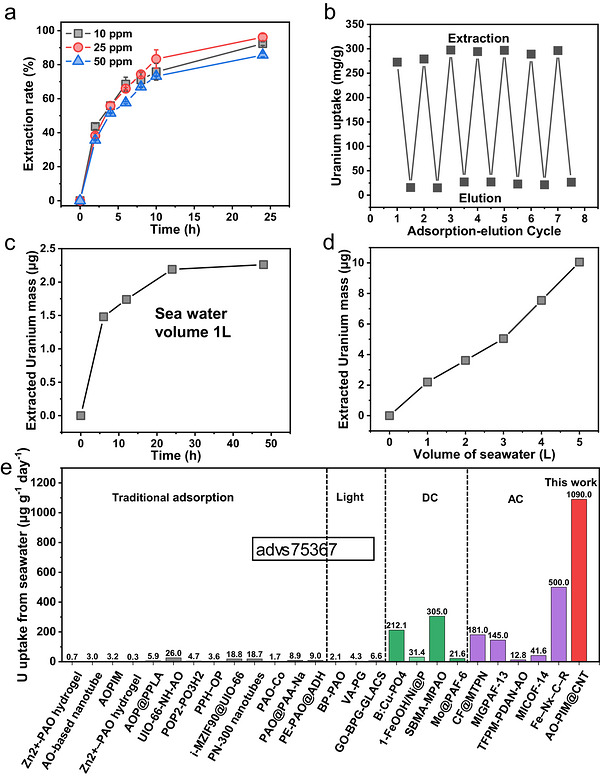
Uranium harvesting from natural seawater via AOPIM‐CNT electrodes. (a) Time‐dependent uranium extraction rates under varying concentrations of uranium‐spiked seawater; (b) Uranium extraction performance over multiple elution cycles from uranium‐spiked seawater; (c) Time‐dependent uranium extraction mass from 1 L unspiked natural seawater; (d) Uranium extraction mass per seawater volume from unspiked natural seawater; (e) Performance comparison with reported systems for uranium extraction from 1 L unspiked natural seawater (detailed data provided in Table S3) [16, 24, 26, 28, 33, 34, 38, 48, 49, 50, 51, 52, 53, 54, 55, 56, 57, 58, 59, 60, 61, 62, 63, 64, 65, 66, 67].

The electrodes were next evaluated in real seawater environments. In U‐spiked seawater (10~50 ppm), the AOPIM‐CNT electrode achieved > 80% extraction efficiency within 24 h (Figure 5a), despite the increased complexity of competing ions. Elution studies revealed that uranium adsorbed from pure water could be completely desorbed using 0.5 M NaHCO_3_ as the eluent, while only ∼50% elution was possible from real seawater due to co‐precipitation with other ions (Figure S32). In contrast, 0.1 M HCl facilitated nearly complete elution, and the electrode's performance was restored through subsequent regeneration in a NaOH solution, demonstrating stability over at least seven elution‐regeneration cycles (Figure 5b) [46, 47]. The performance of the uranium extraction system was ultimately evaluated against the most formidable challenge in this field: the ultralow inherent concentration of uranium in natural seawater (∼3.3 µg L^−^^1^). This constraint makes the mass recovered per unit volume a definitive metric for assessing efficacy. Using a minimal 2 mg microelectrode in unspiked seawater, 2.19 µg of uranium was extracted from 1 L within 24 h, with a slight increase to 2.26 µg after 48 h (Figure 5c). Furthermore, the system demonstrated robust sustainability, achieving a total extraction of 10.05 µg from 5 L of seawater over a continuous 5 day operation with daily replenishment (Figure 5d). Benchmarking against reported values (Figure 5e; Table S3) highlights that the AOPIM‐CNT electrode achieves an exceptional extraction rate of 1090 µg g^−1^ day^−1^ from 1 L natural seawater, surpassing state‐of‐the‐art uranium extraction systems.

## 3 Conclusion

In this work, we developed a hierarchically porous AOPIM‐CNT composite electrode and integrated it with half‐wave rectified alternating current electrochemistry (HW‐ACE) to enable efficient and continuous uranium extraction from seawater. The refined porous architecture of the electrode facilitates rapid uranyl ion diffusion and adsorption, while the amidoxime‐functionalized PIM‐1 ensures strong binding affinity. The incorporation of hydroxylated carbon nanotubes enhances electrical conductivity, enabling the swift electrochemical reduction of adsorbed uranyl ions into crystalline (UO_2_)O_2_·2H_2_O, which subsequently detaches from the electrode surface, thereby regenerating active adsorption sites for repeated use. This adsorption‐promoted electrodeposition mechanism overcomes the limitations of traditional adsorption‐based systems by enabling a fast, continuous, and non‐saturating uranium extraction process. The AOPIM‐CNT system demonstrated exceptional extraction capacity and excellent cycling performance in natural seawater, marking a significant advancement toward practical electrochemical uranium harvesting technologies.

## 4 Experimental Methods

### 4.1 Fabrication of AOPIM‐CNT Electrode

Carbon paper was cut into rectangles as substrates for electrodes. AOPIM‐1 was dissolved in N‐Methyl‐2‐pyrrolidinone (NMP) at 10 wt.%. Different contents of CNTs were added, ranging from 0 to 40 wt.% of AOPIM‐1 mass (see Table S1 for details). The mixtures were blended via ultrasonication and stirring to form cathode slurries, which were coated onto carbon paper substrates. After soaking in ethanol for 1 h and deionized water for 24 h, phase inversion occurred, and the cathodes were dried at 65°C. Each electrode had an active area of 20 mm × 20 mm and a mass of around 9–10 mg.

### 4.2 Uranium Extraction from Uranium Spiked Water

The uranium feed solution was prepared by dissolving uranyl nitrate hexahydrate in deionized water and had a natural pH of ∼4.5 prior to any adjustments. In the HW‐ACE extraction, an AOPIM‐CNT electrode (9~10 mg) was used as the cathode, with an activated carbon electrode as the anode. Both electrodes were positioned parallel, with the active material facing inward. An AC voltage of −5 to 0 V at 400 Hz was applied. For each experiment, 50 mL of the uranium solution was used. Uranium concentration was determined through UV–vis absorption spectra (Shimadzu, UV‐2700, Japan), based on the 652 nm peak for the complexation between Arsenazo (III) and uranyl. The theoretical uranium uptake capacity of the electrode was tested by continuously adding 200 mL of 50 ppm uranium solution every 6 h.

### 4.3 Uranium Extraction From U‐spiked Natural Seawater

The uranium adsorption performance of the AOPIM‐CNT electrode in real seawater was evaluated using spiked seawater from the Bohai Sea, filtered through a 0.22 µm filter. For extraction experiments, 50 mL of 10, 30, and 50 ppm uranium‐spiked seawater were used without cleaning or replacing the electrode. In elution cycling experiments, 50 mL of 50 ppm was used.

### 4.4 Uranium Extraction From Unspiked Natural Seawater

Uranium was extracted from natural seawater filtered through a 0.22 µm filter, using AOPIM‐CNT as the cathode and activated carbon as the anode. Each electrode had an active area of 10 mm × 10 mm and a mass of around 2 mg. To minimize interference, uranium eluted from each electrode was measured separately. Electrodes were immersed in 0.5 M HCl for 48 h, filtered through a 0.22 µm filter, and the uranium concentration was determined by inductively coupled plasma mass spectrometry (ICP‐MS, iCAPTM Qc).

## Conflicts of Interest

The authors declare no conflicts of interest.

## Supporting information




**Supporting File**: advs75367‐000‐SuppMat.docx.

## Data Availability

The data that support the findings of this study are available from the corresponding author upon reasonable request.
