# Supplementary material for: Ultrafast, High‐Capacity Uranium Harvesting From Seawater via a Hierarchically Porous Polymer Electrode
Source: Adv Sci (Weinh). 2026 Apr 20;13(39):e75367. doi: 10.1002/advs.75367 (PMC13335493; doi:10.1002/advs.75367)
Supplement: Supplementary file 1 — Supporting File: advs75367‐000‐SuppMat.docx. [file ADVS-13-e75367-s001.docx]

Supporting Information

**Ultrafast, High-Capacity Uranium Harvesting from Seawater via a Hierarchically Porous Polymer Electrode**

Song *et al.*

Table of Contents

[1.Materials and methods 1](#_Toc226011347)

[1.1 Materials 1](#_Toc226011348)

[1.2 Fabrication 1](#_Toc226011349)

[1.2.1 Fabrication of PIM-1 and AOPIM-1 polymers 1](#_Toc226011350)

[1.2.2 Fabrication of cathode 2](#_Toc226011351)

[1.2.3 Fabrication of anode 3](#_Toc226011352)

[1.2.4 Fabrication of PAO electrode 3](#_Toc226011353)

[1.3 Characterization methods 4](#_Toc226011354)

[1.3.1 Chemical characterization 4](#_Toc226011355)

[1.3.2 Physical characterization 4](#_Toc226011356)

[1.3.3 Electrochemical characterization 5](#_Toc226011357)

[1.4 Electrode performance measurement 6](#_Toc226011358)

[1.4.1 Uranium extraction from uranium spiked water 6](#_Toc226011359)

[1.4.2 Uranium extraction from uranium spiked water with different pH values 7](#_Toc226011360)

[1.4.3 Ionic selectivity experiment 8](#_Toc226011361)

[1.4.4 Stability experiment of uranium extraction performance 8](#_Toc226011362)

[1.4.5 Uranium extraction from unspiked natural seawater 9](#_Toc226011363)

[2.Supplementary figures and tables 10](#_Toc226011364)

[Figure S1. Synthesis route of PIM-1. 10](#_Toc226011365)

[Figure S2. Synthesis route of AOPIM-1. 11](#_Toc226011366)

[Figure S3. ^1^H NMR spectra of (a) PIM-1 and (b) AOPIM-1. 12](#_Toc226011367)

[Figure S4. Pore size distribution of AOPIM-1. 13](#_Toc226011368)

[Figure S5. Schematic representation for the influence of different electrode fabrication methods on ions diffusion. 14](#_Toc226011369)

[Figure S6. Water uptake of electrodes prepared from different membrane fabrication methods. 15](#_Toc226011370)

[Figure S7. Equivalent circuit used to calculate AOPIM-CNT electrode resistance. 16](#_Toc226011371)

[Figure S8. SEM images of activated carbon electrode. 17](#_Toc226011372)

[Figure S9. Optical photos of (a) AOPIM-CNT electrode and (b) activated carbon electrode. 18](#_Toc226011373)

[Figure S10. Photo and Schematic images of the device for uranium extraction via electrodeposition. 19](#_Toc226011374)

[Figure S11. Schematic representation for the half-wave rectified alternating current (HW-ACE). (a) Waveform of HWACE; (b) the process of uranium adsorption and electrochemical precipitation on electrodes under HW-ACE. 20](#_Toc226011375)

[Figure S12. The influence of different voltage on uranium extraction performance. (a) The variation of uranium extraction rate over time under different voltages; (b) Uranium uptake within 5 hours under different voltages. 21](#_Toc226011376)

[Figure S13. Amperometric i-t Curves under 0~5 V square waves with different frequencies: (a-b) 0 Hz, (c-d) 100 Hz, (e-f) 200 Hz, (g-h) 400 Hz. 22](#_Toc226011377)

[Figure S14. Uranium extraction performance under. (a) The variation of uranium extraction rate of electrodes under different external fields in uranium spiked DI-water over time; (b) Uranium uptake of electrodes under different external fields in uranium spiked DI-water over time; (c) The variation of uranium extraction rate of electrodes under different external fields in uranium spiked sea water over time; (d) Uranium uptake of electrodes under different external fields in uranium spiked sea water over time. 23](#_Toc226011378)

[Figure S15. The linear regression fitting of concentration-absorbance of (a) DI water and (b) natural seawater with different uranium concentrations. 24](#_Toc226011379)

[Figure S16. FTIR spectra of AOPIM-CNT composite electrodes with varying CNTs content. 25](#_Toc226011380)

[Figure S17. SEM images of electrodes with varying CNTs content. 26](#_Toc226011381)

[Figure S18. SEM images of AO-PIM-40%CNTs electrode. 27](#_Toc226011382)

[Figure S19. Surface area of electrodes with varying CNTs content. 28](#_Toc226011383)

[Figure S20. The variation of uranium extraction rate over time for electrodes with varying CNTs content. 29](#_Toc226011384)

[Figure S21. (a) Synthesis route of PAO; (b) FTIR spectra of PAN and PAO polymer. 30](#_Toc226011385)

[Figure S22. Uranium extraction capacities of different electrodes and voltages within 5 hours. 31](#_Toc226011386)

[Figure S23. SEM image of the AOPIM-CNT electrode after the electrochemical uranium extraction and its corresponding elemental mapping. 32](#_Toc226011387)

[Figure S24. EDS spectra of AOPIM-CNT electrode after the electrochemical uranium extraction. 33](#_Toc226011388)

[Figure S25. The zeta potential of (a) AO-PIM-10%CNT electrode and (b) AOPIM-1. 34](#_Toc226011389)

[Figure S26. The existence species of uranium ions under different pH conditions. 35](#_Toc226011390)

[Figure S27. The influence of different pH conditions on the uranium extraction performance of electrodes. 36](#_Toc226011391)

[Figure S28. Ion selectivity of AOPIM-CNT electrode. 37](#_Toc226011392)

[Figure S29. (a) CV curves in NaCl solution; (b) CV curves in seawater; (c) The variation of uranium extraction rate over time under different voltages. 38](#_Toc226011393)

[Figure S30. (a) XRD spectra of uranyl nitrate; (b) Raman spectra of uranyl nitrate at 532 nm wavelength; (c) Raman spectra of pristine AOPIM-CNT and electrode after electrochemical precipitation at 532 nm wavelength; (d) Quasi-in situ Raman spectra of electrode after 48 hours of uranium extraction under different electric field strengths; (e) XPS survey spectrum of AOPIM-CNT after the uranium extraction; (f) high-resolution U 4*f* spectra of AOPIM-CNT after the uranium extraction. 39](#_Toc226011394)

[Figure S31. Uranium extraction capacities in 24 hours under different concentrations of uranium-spiked seawater. 40](#_Toc226011395)

[Figure S32. AOPIM-CNT electrode uranium uptake and elution with NaHCO_3_ in (a) 50 ppm uranium-spiked fresh water and (b) seawater with varying uranium concentrations. 41](#_Toc226011396)

[Figure S33. Optical photos of real seawater uranium extraction equipment, and working electrode before and after extraction. 42](#_Toc226011397)

[Table S1. Formulation details of various AOPIM-CNT electrode slurry. 43](#_Toc226011398)

[Table S2. Concentration of different elements 44](#_Toc226011399)

[Table S3. Performance comparison with reported systems for uranium extraction from unspiked natural seawater. 45](#_Toc226011400)

[References 47](#_Toc226011401)

# 1.Materials and methods

## **1.1 Materials**

5,5′,6,6′-tetrahydroxy-3,3,3′,3′-tetramethylspirobisindane (TTSBI, purity: 99%), tetra-fluoroterephthalonitrile (TFTPN, purity: 99%), Extra dry N-Methyl-2-pyrrolidinone (NMP, water < 50 ppm, purity: 99.5%), Uranyl Nitrate Hexahydrate (purity: 99%), Arsenazo III (purity: 99%), polyvinylidene fluoride (PVDF, Solef 1015, Mn: 238000) and hydroxylamine aqueous solution (purity: 50 wt%) were purchased from Aladdin Co. Ltd., China. K_2_CO_3_ (purity: 99%) were purchased from Sigma-Aldrich. NaOH (purity: 99%), hydrochloric acid (Mass fraction 37%), Carbon black (ECP600JD, purity: 99%), NaCl (purity: 99%), NaHCO_3_ (purity: 99%), Na_2_CO_3_ (purity: 99%), FeCl_3_ (purity: 99%), Na_3_VO_4_ (purity: 99%), Anhydrous ethanol (purity: 99%), N,N-dimethyl formamide (DMF, purity: 99%), Poly acrylonitrile (PAN, Mw 15000) and tetrahydrofuran (THF, purity: 99%) were purchased from Sinopham Chemical Reagent Co. Ltd., China. Toluene (purity: 99%) was purchased from Alfa Aesar, UK. Hydrophilic modified single-walled carbon nanotubes (CNTs, purity:90%, diameter: 1~2 nm, hydroxyl group: 3.96 wt%) was purchased from Nanjing XFNANO Materials Tech Co., Ltd., China. Activated carbon (AC, YP-50F) was purchased from Foshan Porous Carbon Tech Co., Ltd., China. Carbon paper (HCP020N) was purchased from Suzhou Sinero Technology Co., Ltd.

## **1.2 Fabrication**

## 1.2.1 Fabrication of PIM-1 and AOPIM-1 polymers

Polymer of intrinsic microporosity (PIM-1) was obtained following a previously reported method. 3.001 g (15 mmol) TFTPN, 5.106 g (15 mmol) TTSBI and 20 mL NMP were added into a 100 mL three-necked flask. After being completely dissolved, 6.21 g (45 mmol) anhydrous milled K_2_CO_3_ was added and the flask was placed into a 140 °C oil bath under mechanical stirring and a nitrogen atmosphere. After approximately 3 minutes, a viscous yellow solution formed, and 10 mL of toluene was added. Several minutes later, when the solution becomes viscous again, a further 10 mL of toluene was added to dilute the solution. The above operation was repeat until the reaction takes half an hour. Then, the mixture was slowly poured into 400 mL methanol, and a bright yellow filamentous polymer was observed. The polymer product was dissolved in chloroform and reprecipitated in methanol, and then refluxed in a solution containing a small amount of hexane in a 1:1 ratio of water to ethanol for 4-5 h and dried at 80 °C under vacuum for 48 h.

Hydrophilic amidoxime modified PIM-1 (AOPIM-1) was synthesized by dissolving 3 g PIM-1 in 150 mL THF and heating to around 65 °C under magnetic stirring and a nitrogen atmosphere. Then, 30.0 mL hydroxyamine aqueous solution (50 wt%) was added dropwise to avoid the formation of white precipitate, and the solution was further refluxed for 20 hours. The mixture was rotary evaporated until viscous, then slowly poured it into a 1 L mixture of ethanol and water, and white polymer was observed. The polymer was washed thoroughly with ethanol, hexane and water, and then dried at 110 °C for 24 h.

### 1.2.2 Fabrication of cathode

Carbon paper was cut into rectangles as substrate for electrodes. AOPIM-1 was dissolved in NMP to prepare a solution with a 10 wt% concentration of AOPIM. Then, CNTs with varying mass fractions were added, with CNTs accounting for 0 wt%, 2 wt%, 5 wt%, 10 wt%, 20 wt% and 30 wt% of the mass of AOPIM-1 (Detailed data can be found in Table S1). The mixture was uniformly blended through repeated ultrasonication and stirring to obtain the cathode slurry. The cathode was obtained by slurry coating the cathode slurry on a carbon paper substrate, then soaked in anhydrous ethanol for 1 hour, followed by soaking in deionized water for 24 h to finish phase inversion, and dried at 65 °C. The active component of each electrode was formed into a square shape of 20 mm × 20 mm, with a mass of approximately 9~10 mg. The electrode obtained by water induced phase inversion was made by soaking coated electrode in deionized water for 24 h and dried at 65 °C. The electrode obtained by solvent evaporation was made by placing the coated electrode on a heating table at 65 °C for more than 6 hours to evaporate the solvent completely.

### 1.2.3 Fabrication of anode

The anode used the commonly employed an activated carbon electrode as described in the literature. Carbon paper was cut into rectangles as electrode substrates. PVDF, AC and CB were suspended into NMP solvent at a mass ratio of 2:7:1. The mixture was prepared into a uniform slurry through repeated ultrasonication and stirring. The anode was obtained by slurry coating the slurry on a carbon paper substrate, then placed it on a heating table at 65 °C to evaporate the solvent completely.

### 1.2.4 Fabrication of PAO electrode

The purchased 1 g PAN powder was added to 9 g DMF and stirred at 60 °C for 12 h to fully dissolve it, and then mixed with 0.1 g CNTs. The mixture was uniformly blended through repeated ultrasonication and stirring to obtain the cathode slurry. The cathode was obtained by slurry coating the cathode slurry on a carbon paper substrate, then soaked in anhydrous ethanol for 1 hour, followed by soaking in deionized water for 24 h to finish phase inversion, and dried at 65 °C. The obtained PAN-CNT electrode was treated with an aqueous solution containing 80 mg L^-1^ hydroxylamine and 60 mg L^-1^ Na_2_CO_3_ at 70  °C for 2 h under gentle stirring. After the reaction, the resulting product was thoroughly rinsed with deionized water several times and dried at 65  °C for 24 h to obtain the PAO-CNT electrode.

## **1.3 Characterization methods**

### 1.3.1 Chemical characterization

The chemical structure of the polymer was analyzed by ^1^H nuclear magnetic resonance (NMR) on a Bruker 400 MHz spectrometer, and the solvent for PIM-1 and AOPIM-1 is chloroform-d (CDCl_3_) and dimethyl sulfoxide-d_6_ (DMSO-d6) respectively. Fourier Transform infrared spectroscopy (FTIR) spectra of electrodes were obtained using a Nicolet 6700 FTIR spectrometer (USA). Raman spectra were conducted using a 532 nm excitation laser by inVia Qontor Laser confocal raman microspectroscopy (Renishaw, HongKong). For the quasi-in-situ Raman analysis, electrodes from the same batch were employed to extract uranium from a 500 mL, 100 ppm uranium-spiked solution under various applied voltages for 48 hours. X-ray photoelectron spectroscopy (XPS) was conducted using a ESCALAB 250Xi (Thermo Scientific, USA).

### 1.3.2 Physical characterization

X-ray Diffraction (XRD) patterns were obtained using an X-ray diffractometer (D8 Advance, Germany). The morphology of the as-prepared electrode was observed using a field emission scanning electron microscope (SEM) (Hitachi S8230, Japan). In addition, the elemental distribution of electrode surface was obtained by energy dispersive spectroscopy (EDS) on this instrument. Before capturing SEM image and EDS data, a thin layer of Pt was sputtered onto the electrode under 10 mA for 120 s (GVC-2000). Nitrogen adsorption/desorption measurements were performed on a Quantachrome Autosorb IQ-MP-MP at 77 K. All samples were degassed at 120 °C for 24 h before nitrogen adsorption measurements were performed. The surface charge of the membrane was determined by streaming potential measurement using a SurPASS 3 electrokinetic analyzer with a flat-plate measuring cell (10 mm × 20 mm). The pore size distribution of electrode was measured by Beishide 3H-2000PB filter membrane pore size analyzer，and the infiltration solution was anhydrous ethanol. The porosity was obtained by measuring the water content of the materials and calculated by through Eq. (1):

$P=\frac{m_{w}-m_{d}}{m_{w}}$ (1)

where P is the porosity of AOPIM-CNT electrodes, m_w_ is the mass of the electrode after immersion by water (surface dried), m_d_ is the mass of the electrode after drying. The thermogravimetric analysis (TGA) of the membrane was obtained by Netzsch TG209 F3, all samples were initially conditioned at 40 °C for under a nitrogen flow of 60 mL min^−1^ to remove the moisture and then they were heated from 40 to 800 °C at a ramping rate of 5 °C min^-1^.

### 1.3.3 Electrochemical characterization

The electrochemical behaviors of AOPIM-CNT electrodes were evaluated with a three-electrode system. The reference electrode is an Ag/AgCl electrode; the counter electrode is an activated carbon electrode with a size of 20 mm × 20 mm. Amperometric i-t Curves were conducted in natural seawater using an electrochemical workstation with the applied voltage set to 0 V and externally coupled with a 0–5 V square wave at various frequencies; the data acquisition interval was 0.001 s. Electrochemical impedance spectroscopy (EIS) measurements were performed in a 50 ppm uranyl nitrate aqueous solution, where the initial voltage was set to the open-circuit potential of the working electrode after stabilization in the solution, and the frequency range was from 0.01 to 100,000 Hz. Cyclic voltammetry (CV) measurements were carried out in 0.1 mol L^-1^ NaCl solution before and after the addition of 50 ppm uranyl nitrate, and seawater before and after the addition of 100 ppm uranyl nitrate, over a potential range of –1.8 V to 0.5 V at a scan rate of 0.1 V s^-1^. The above electrochemical tests collected by a CHI660D electrochemical workstation.

## **1.4 Electrode performance measurement**

### 1.4.1 Uranium extraction from uranium spiked water

Uranium spiked water was made by dissolving Uranyl-Nitrate Hexahydrate into deionized water. During HW-ACE extraction, an AOPIM-CNT electrode (9~10 mg) was used as the negative electrode and activated carbon electrode as the positive electrode. The two electrodes are placed parallel to each other, with the side containing the active material facing inward. In the case of HW-ACE extraction, an a.c. voltage of −5 V to 0 V was used with a frequency of 400 Hz. In each adsorption experiment, 50 mL of spiked uranium solution was used.

The comparative experiments on uranium extraction performance under various driving forces were conducted using 200 mL of a 50 ppm uranium-containing solution, with either a 1 V DC voltage or a 0-5 V, 400 Hz square wave serving as the driving force.

The concentrations of uranium in the solution were determined via ultraviolet-visible (UV-Vis) absorption spectra (Shimadzu, UV-2700, Japan) on the basis of the specific peak at 652 nm for the complexation between Arsenazo(III) chromogenic agent and uranyl. The test sample was prepared by mixing 3 mL of deionized water, 1 mL of 500 ppm Arsenazo aqueous solution, 0.5 mL of 0.1 M HCl, and 0.5 mL of uranium solution. The uranium spiked solutions in pure water backgrounds and real seawater backgrounds use different UV standard curves, which are plotted based on measurements from inductively coupled plasma atomic emission spectroscopy (ICP-OES, Aglient 5100). The uranium loaded on the electrode can be calculated by Eq. (2):

$m_{U}=\left( C_{0}-C_{t} \right)\times V$ (2)

where m_U_ is the adsorption mass of uranium, C_0_ is the original concentration of the solution, C_t_ is the concentration of uranium detected at a specific point t during the whole adsorption and V is the total volume of the solution. The amount of uranium extracted by AOPIM-CNT electrode, m_E_ was determined from the Eq. (3):

$m_{E}=\frac{m_{U}}{w}$ (3) where w is mass of active component of AOPIM-CNT electrode. The uranium extraction rate, R was determined from Eq. (4):

$R=\frac{C_{0}-C_{t}}{C_{0}}$ (4)

### 1.4.2 Uranium extraction from uranium spiked water with different pH values

Uranium spiked water with different pH values were prepared by adding appropriate amounts of HCl or NaOH to a 10 ppm uranyl solution. The initial pH of the unadjusted uranium spiked water was approximately 4.5. All other experimental conditions and procedures remained consistent with those described in the preceding section.

### 1.4.3 Ionic selectivity experiment

The ion selectivity of the electrode was assessed using 60 mL of a mixed solution containing U and representative competing ions (Fe, V), each at concentrations 1000-fold higher than those typically found in seawater (Table S2). To mimic natural seawater conditions, the pH of the mixed solution was adjusted to 8.0 with sodium hydroxide solution. Prior to each experiment, the solution was freshly prepared to prevent hydrolysis upon prolonged standing. The experiments were carried out for 12 hours. Post-reaction, samples were collected and immediately acidified to a pH below 3.0 using dilute hydrochloric acid. Ion concentrations were then determined via inductively coupled plasma optical emission spectrometry (ICP-OES).

### 1.4.4 Stability experiment of uranium extraction performance

The theoretical uranium uptake capacity of the electrode by continuously adding an aqueous solution of uranium. 200 mL of 50 ppm uranium solution was used in each time, and replacing it with a fresh solution every 6 hours.

The uranium adsorption performance of the electrode in a real seawater environment is evaluated by uranium spiked seawater which was made by dissolving Uranyl-Nitrate Hexahydrate into real seawater collected from Bohai sea (China) to different concentrations. The seawater used was filtered through a 0.22 µm filter to remove insoluble impurities. 50 mL of 10 ppm, 30 ppm, and 50 ppm uranium-spiked seawater solutions were continuously used for uranium extraction experiments without cleaning or replacing the electrode in between.

50 mL of uranium spiked seawater was used in the uranium elution cycling experiments on the electrode each cycle, with concentrations of 50 ppm. The ion-loaded electrode was eluted by immersion in 50 mL of 0.1 M HCl solution for 24 h. After elution, the electrode was regenerated in 50 mL of 5 mM NaOH for 1 h and then used for the next cycle. The concentrations of uranium in the solution were determined via UV-Vis absorption spectra.

### 1.4.5 Uranium extraction from unspiked natural seawater

Unspiked real seawater after 0.22 µm filtration was used for uranium extraction experiment in natural seawater. The AOPIM-10 wt% CNTs electrode (~1 cm^2^, 2 mg) was used as the negative electrode and activated carbon electrode (~1 cm^2^, 2 mg) was used as the positive electrode. To mitigate the influence of the overwhelming presence of interfering ions in natural seawater, the mass of uranium eluted from the electrode was measured, and each data was derived from a distinct electrode. Each electrode after uranium extraction was immersed in 20 mL of 0.5 M HCl solution for 48 h. Subsequently, the solution was filtered through a 0.22 µm filtration. The uranium concentration of the resultant solution was measured by inductively coupled plasma mass spectrometry (ICP-MS, iCAPTM Qc).

# 2.Supplementary figures and tables

**
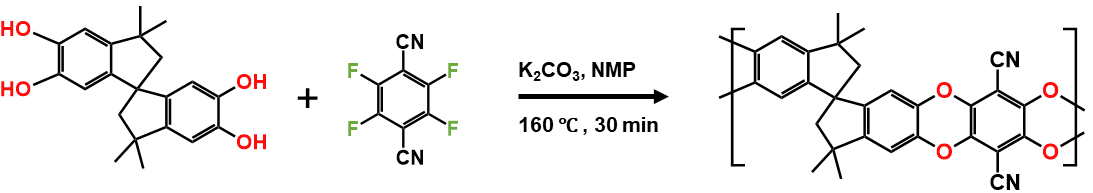
**

## Figure S1. Synthesis route of PIM-1.

## Figure S2. Synthesis route of AOPIM-1.

**
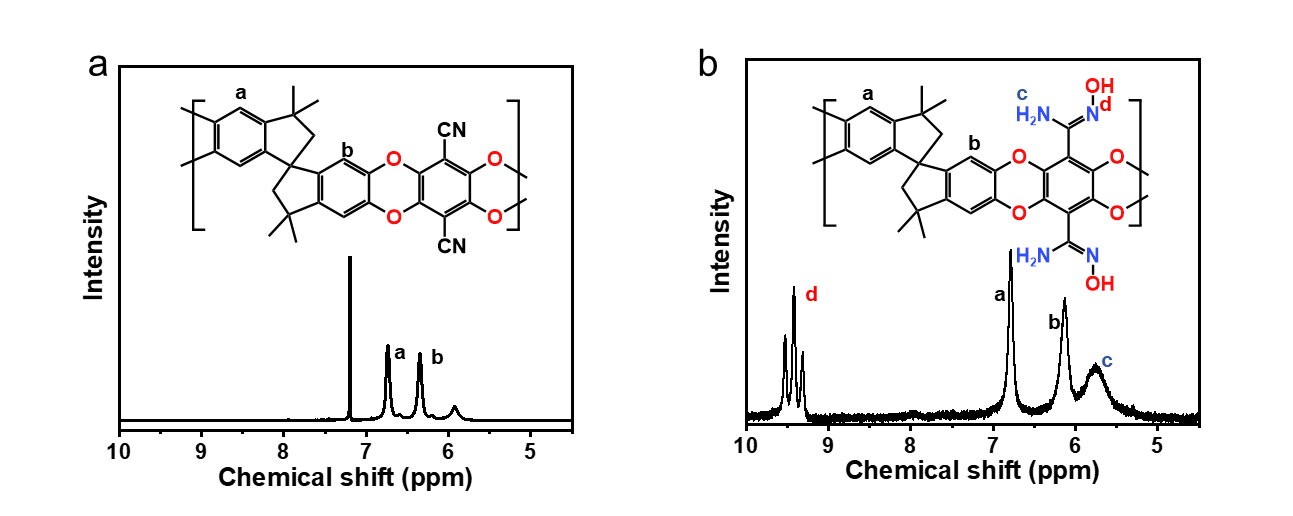
**

## Figure S3. ^1^H NMR spectra of (a) PIM-1 and (b) AOPIM-1.


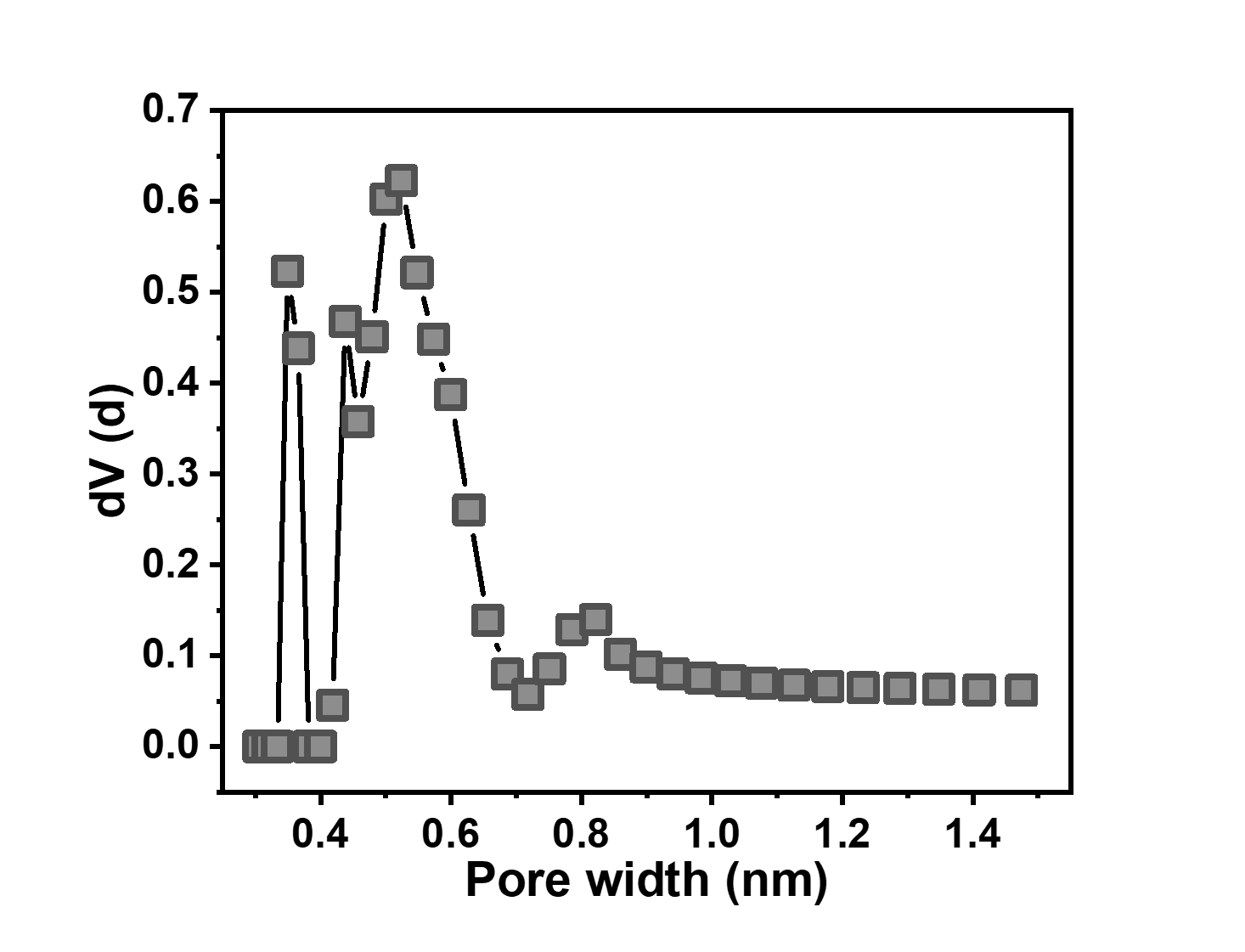


## Figure S4. Pore size distribution of AOPIM-1.

## Figure S5. Schematic representation for the influence of different electrode fabrication methods on ions diffusion.

**
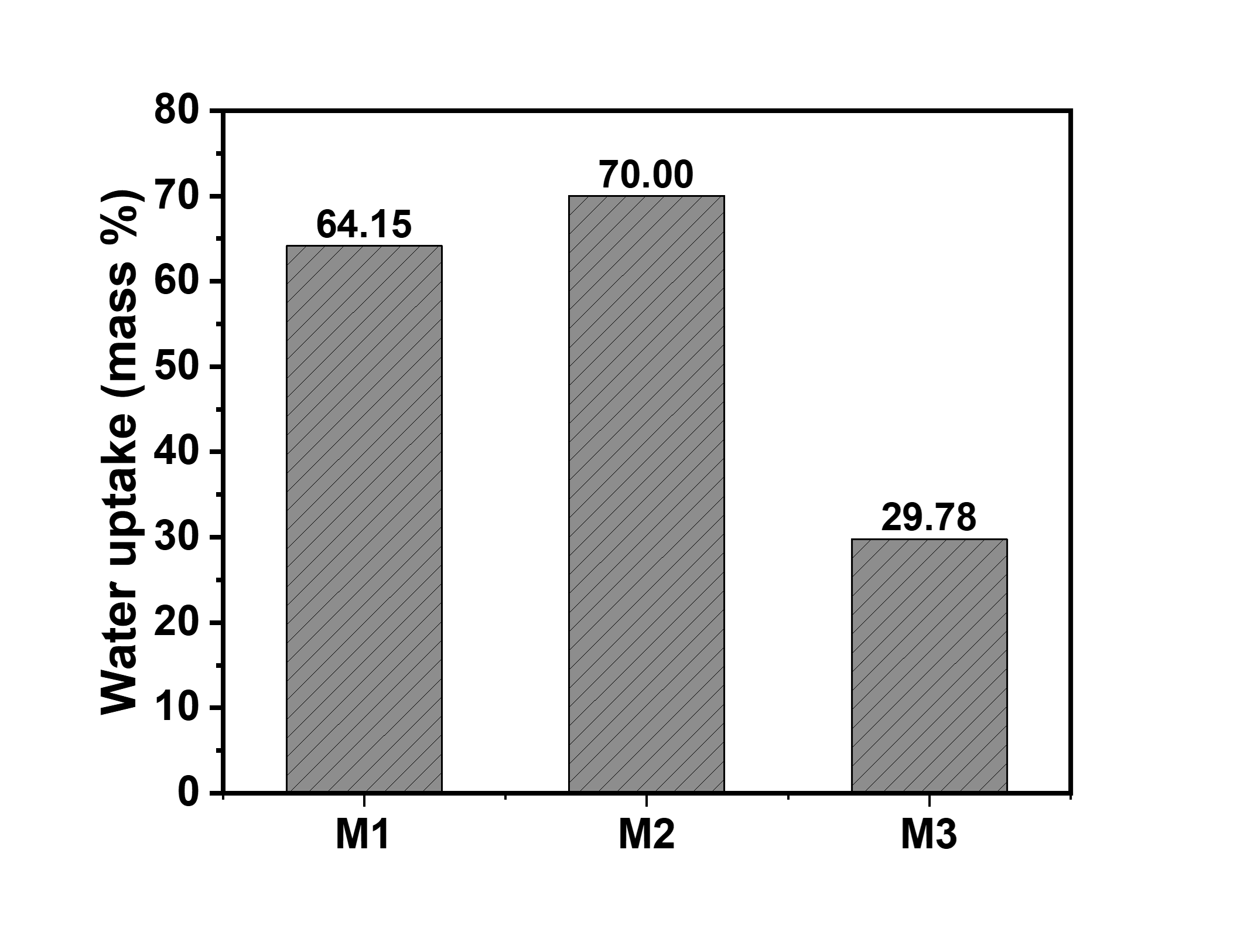
**

## Figure S6. Water uptake of electrodes prepared from different membrane fabrication methods.

## Figure S7. Equivalent circuit used to calculate AOPIM-CNT electrode resistance.

**
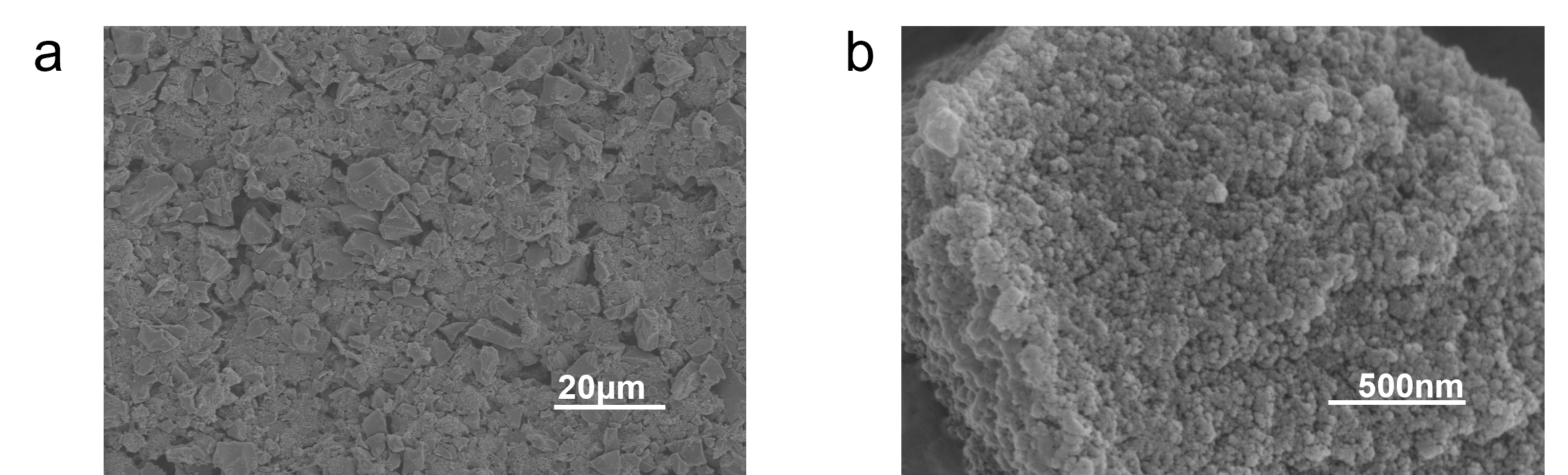
**

## Figure S8. SEM images of activated carbon electrode.

**
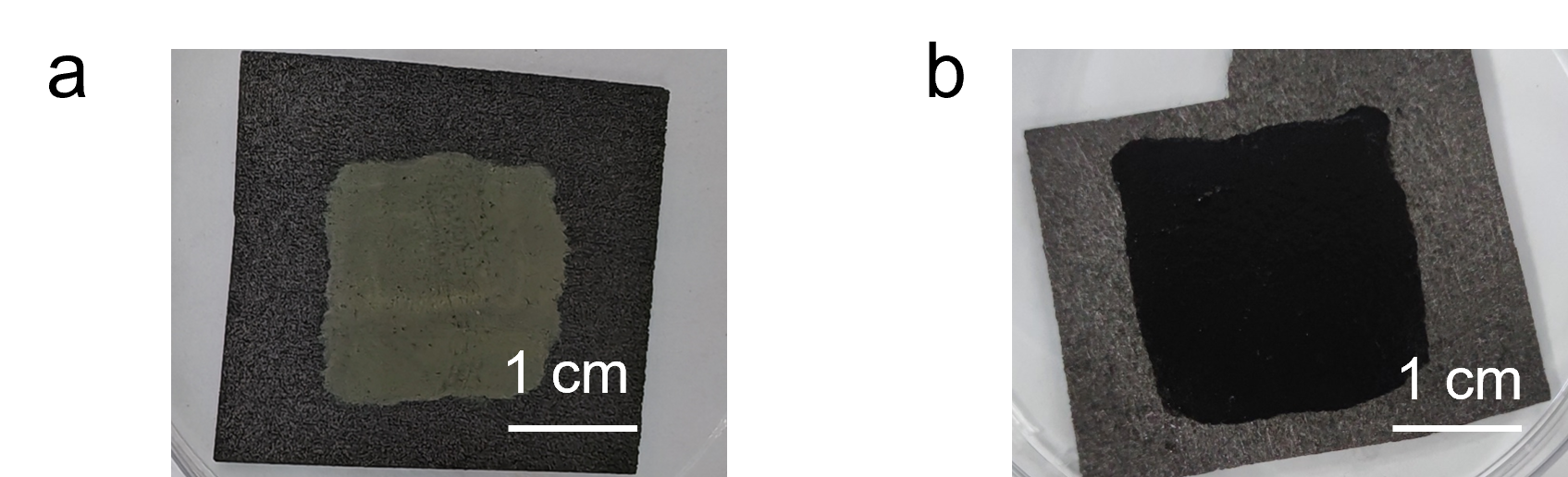
**

## Figure S9. Optical photos of (a) AOPIM-CNT electrode and (b) activated carbon electrode.

**
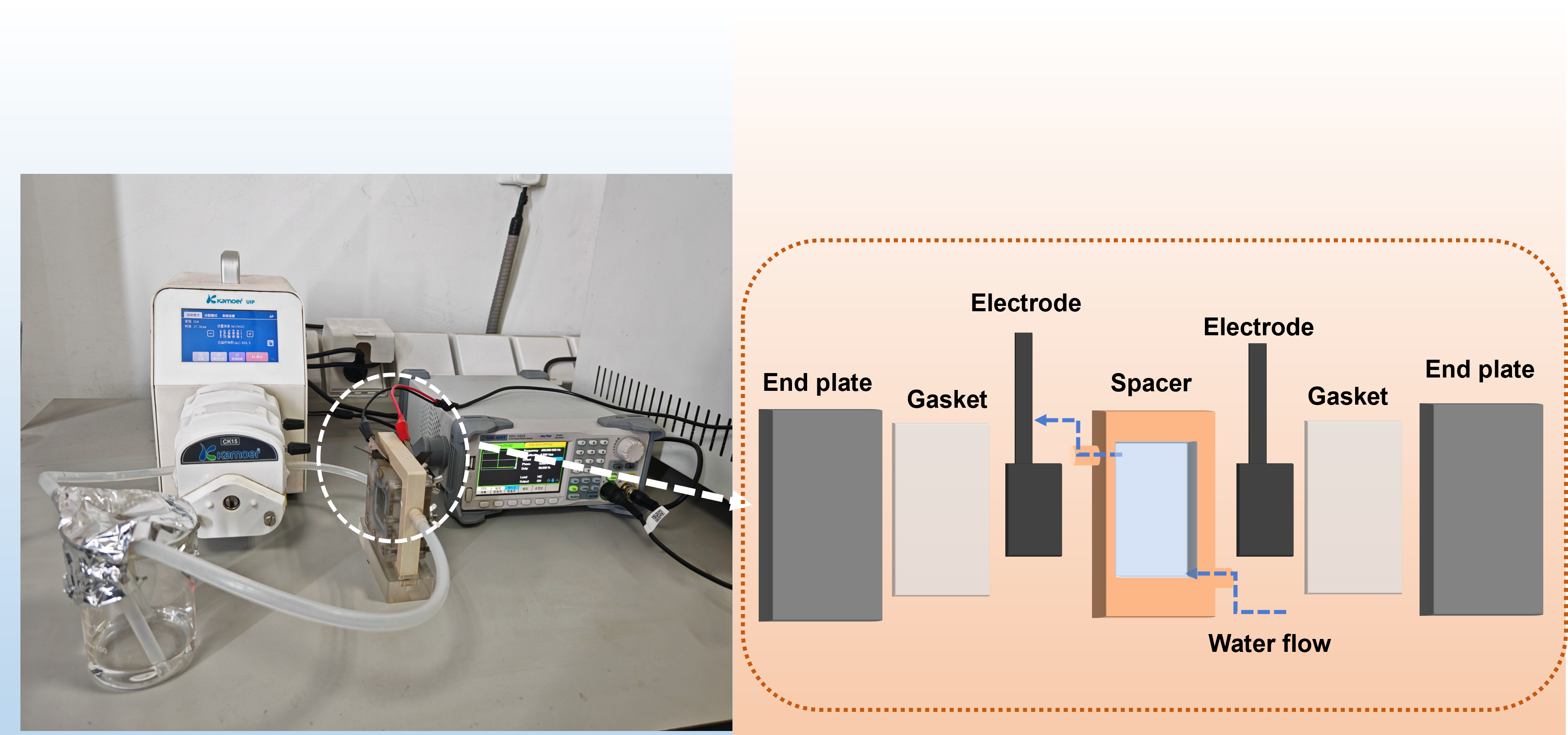
**

## Figure S10. Photo and Schematic images of the device for uranium extraction via electrodeposition.

**
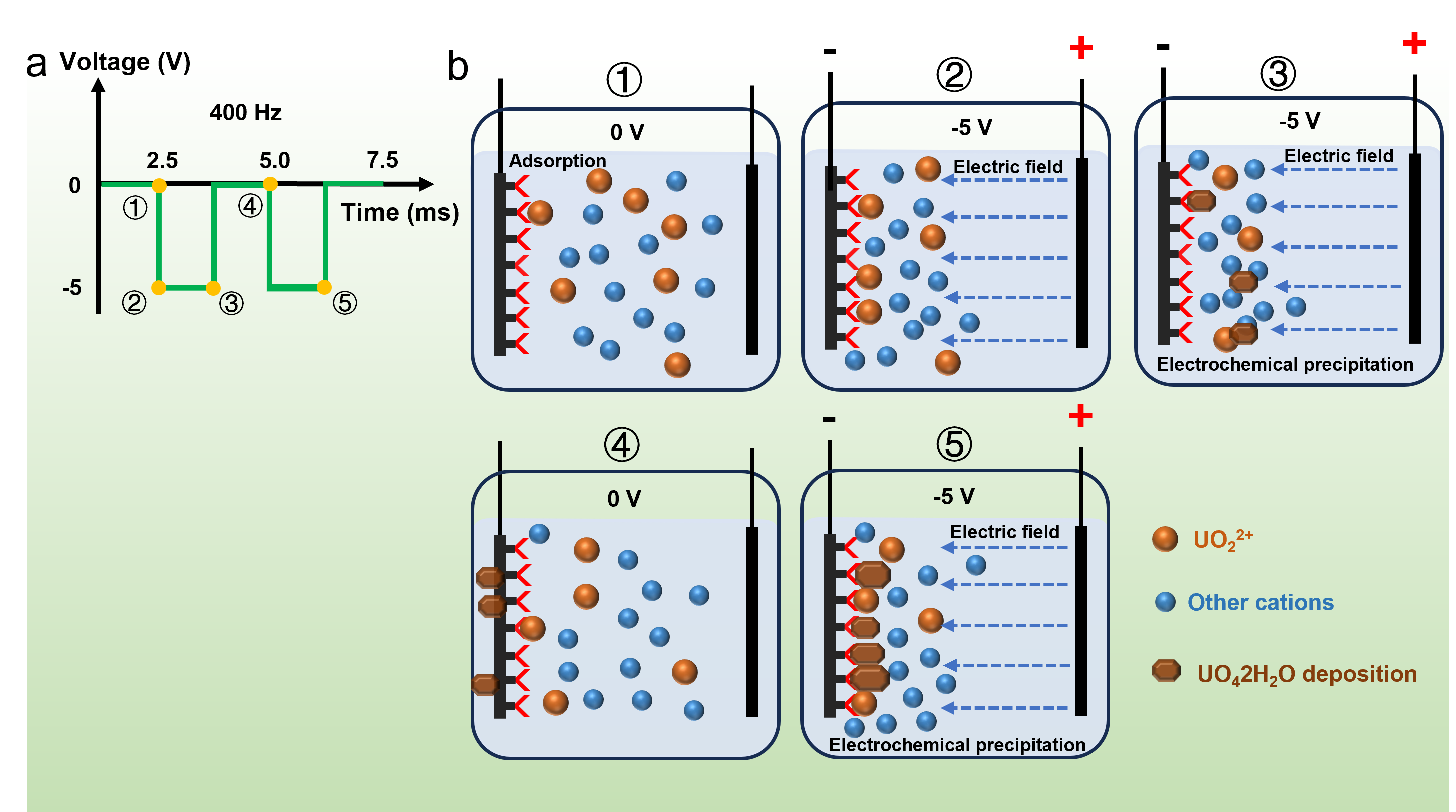
**

## Figure S11. Schematic representation for the half-wave rectified alternating current (HW-ACE). (a) Waveform of HWACE; (b) the process of uranium adsorption and electrochemical precipitation on electrodes under HW-ACE.

## Figure S12. The influence of different voltage on uranium extraction performance. (a) The variation of uranium extraction rate over time under different voltages; (b) Uranium uptake within 5 hours under different voltages.

## Figure S13. Amperometric i-t Curves under 0~5 V square waves with different frequencies: (a-b) 0 Hz, (c-d) 100 Hz, (e-f) 200 Hz, (g-h) 400 Hz.

## Figure S14. Uranium extraction performance under different modes. (a) The variation of uranium extraction rate of electrodes under different external fields in uranium spiked DI-water over time; (b) Uranium uptake of electrodes under different external fields in uranium spiked DI-water over time; (c) The variation of uranium extraction rate of electrodes under different external fields in uranium spiked sea water over time; (d) Uranium uptake of electrodes under different external fields in uranium spiked sea water over time.

## Figure S15. The linear regression fitting of concentration-absorbance of (a) DI water and (b) natural seawater with different uranium concentrations.


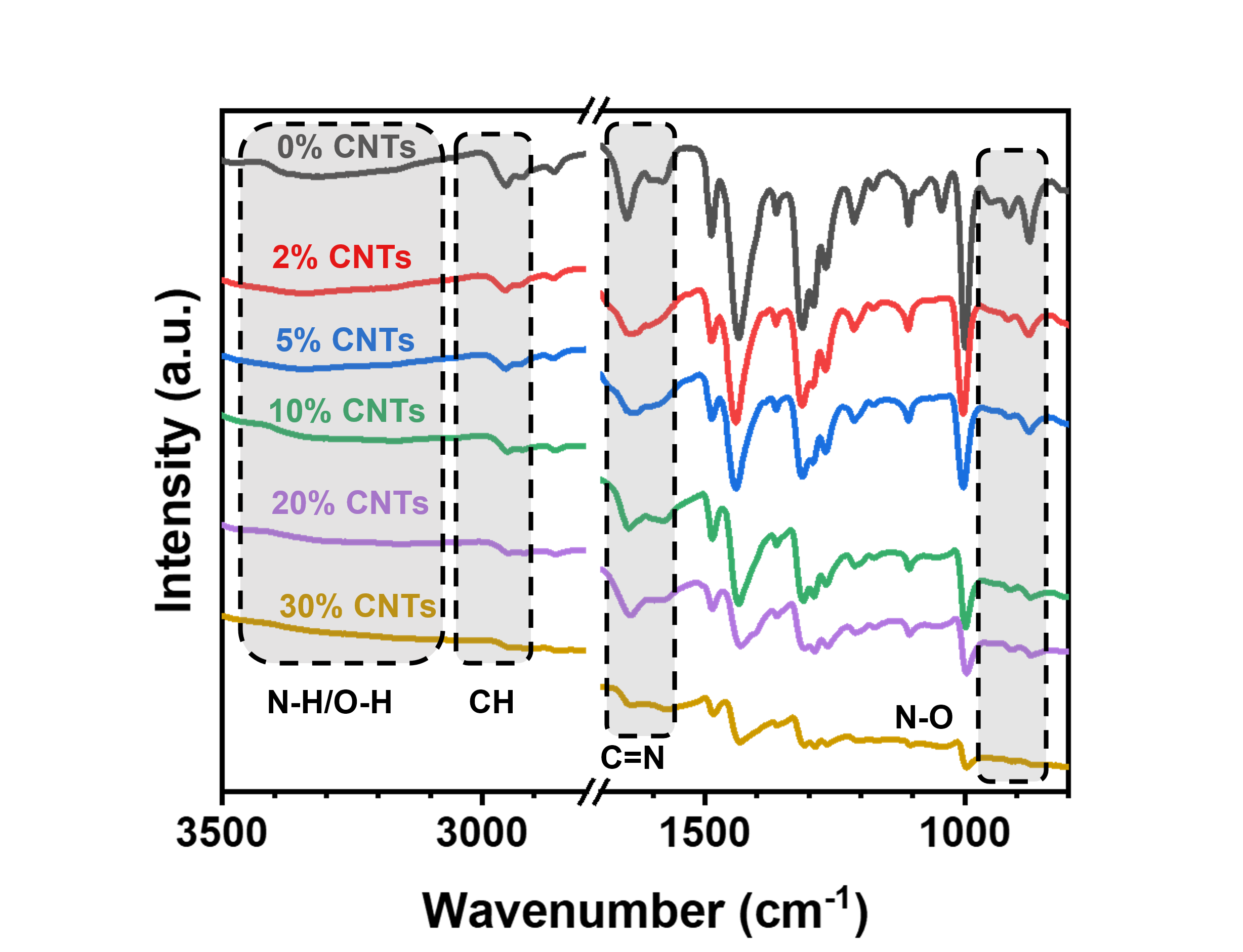


## Figure S16. FTIR spectra of AOPIM-CNT composite electrodes with varying CNTs content.

Figure S17. SEM images of electrodes with varying CNTs content. (a) SEM images of AO-PIM, (b) AO-PIM-2%CNTs, (c) AO-PIM-5%CNTs, (d) AO-PIM-10%CNTs, (e) AO-PIM-20%CNTs, (f) AO-PIM-30%CNTs electrodes.

## Figure S18. SEM images of AO-PIM-40%CNTs electrode.


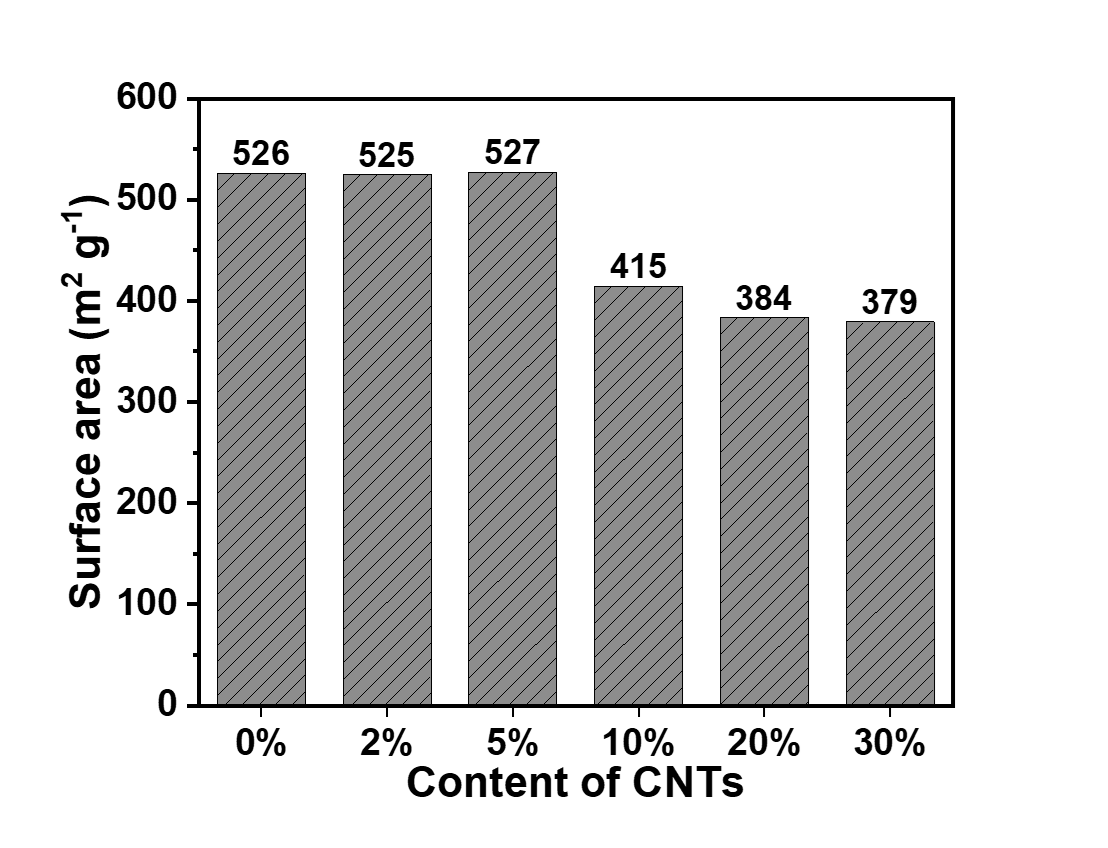


## Figure S19. Surface area of electrodes with varying CNTs content.


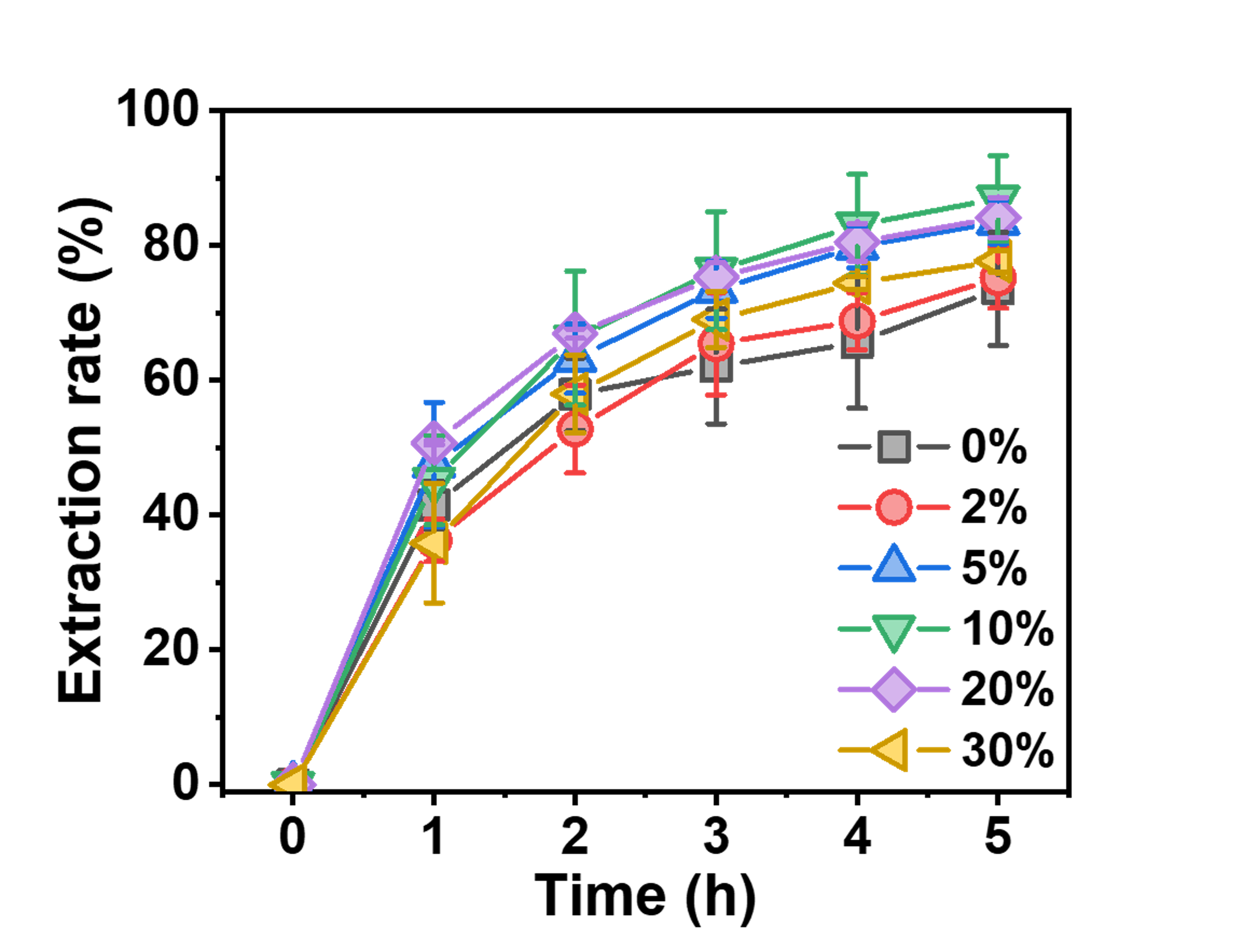


## Figure S20. The variation of uranium extraction rate over time for electrodes with varying CNTs content.

**
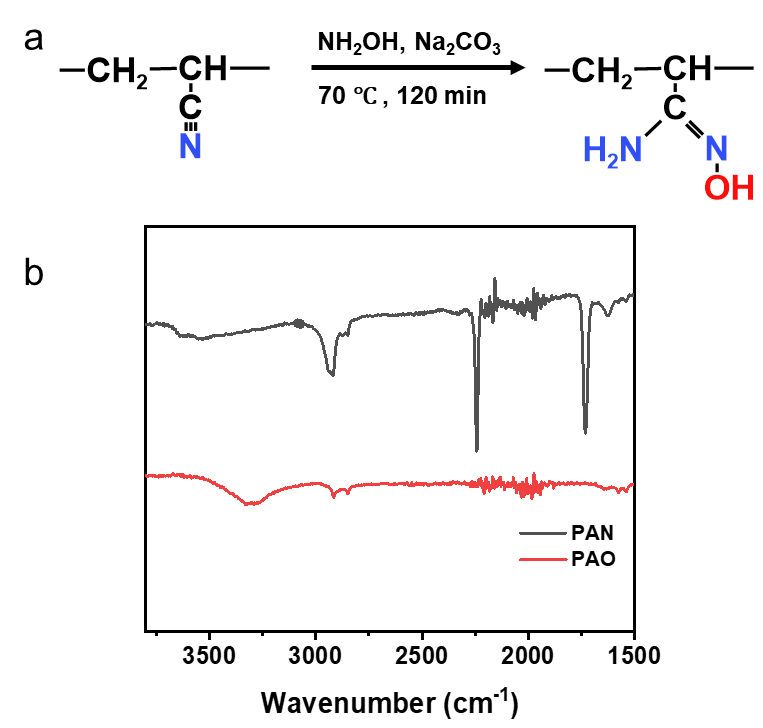
**

## Figure S21. (a) Synthesis route of PAO; (b) FTIR spectra of PAN and PAO polymer.

**
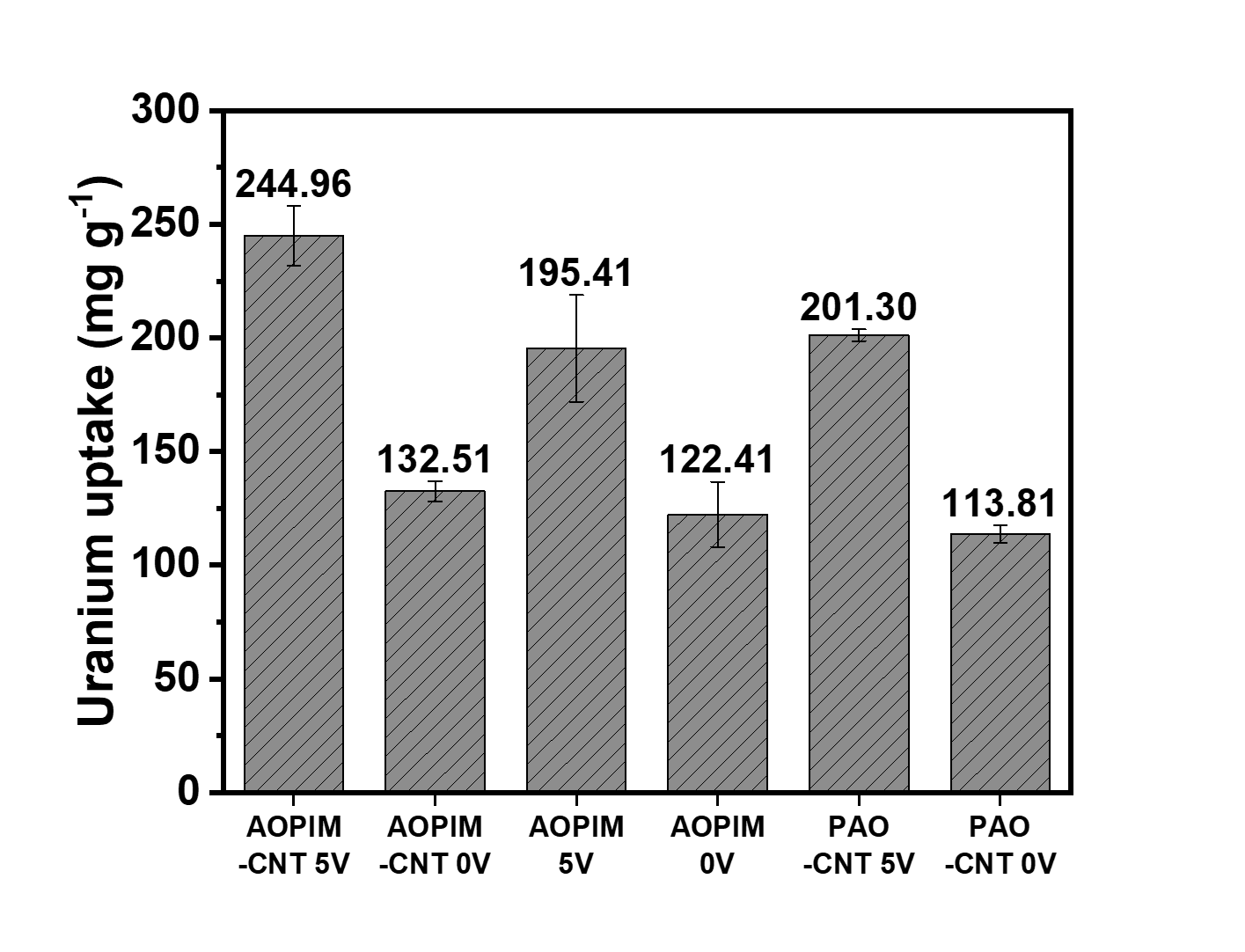
**

## Figure S22. Uranium extraction capacities of different electrodes and voltages within 5 hours.

**
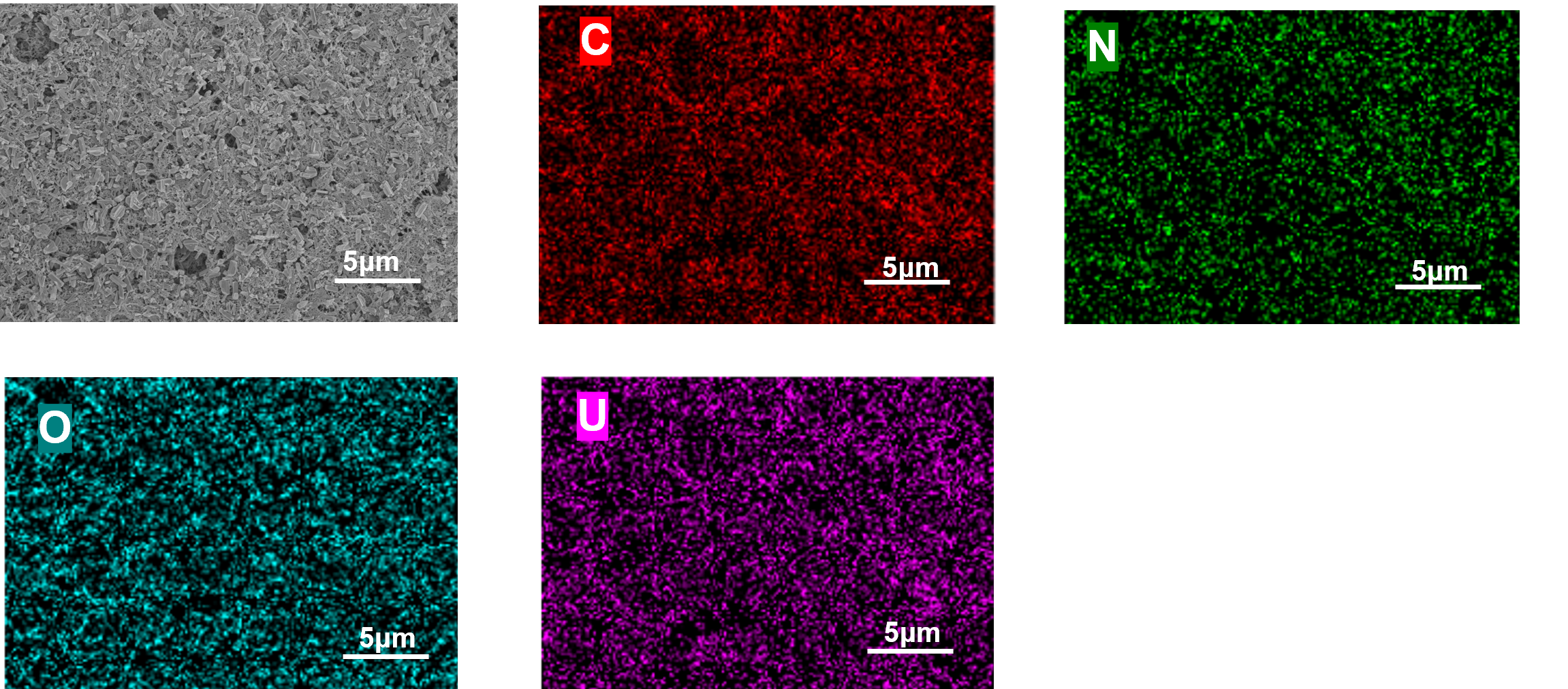
**

## Figure S23. SEM image of the AOPIM-CNT electrode after the electrochemical uranium extraction and its corresponding elemental mapping.

## Figure S24. EDS spectra of AOPIM-CNT electrode after the electrochemical uranium extraction.

## Figure S25. The zeta potential of (a) AO-PIM-10%CNT electrode and (b) AOPIM-1.

**
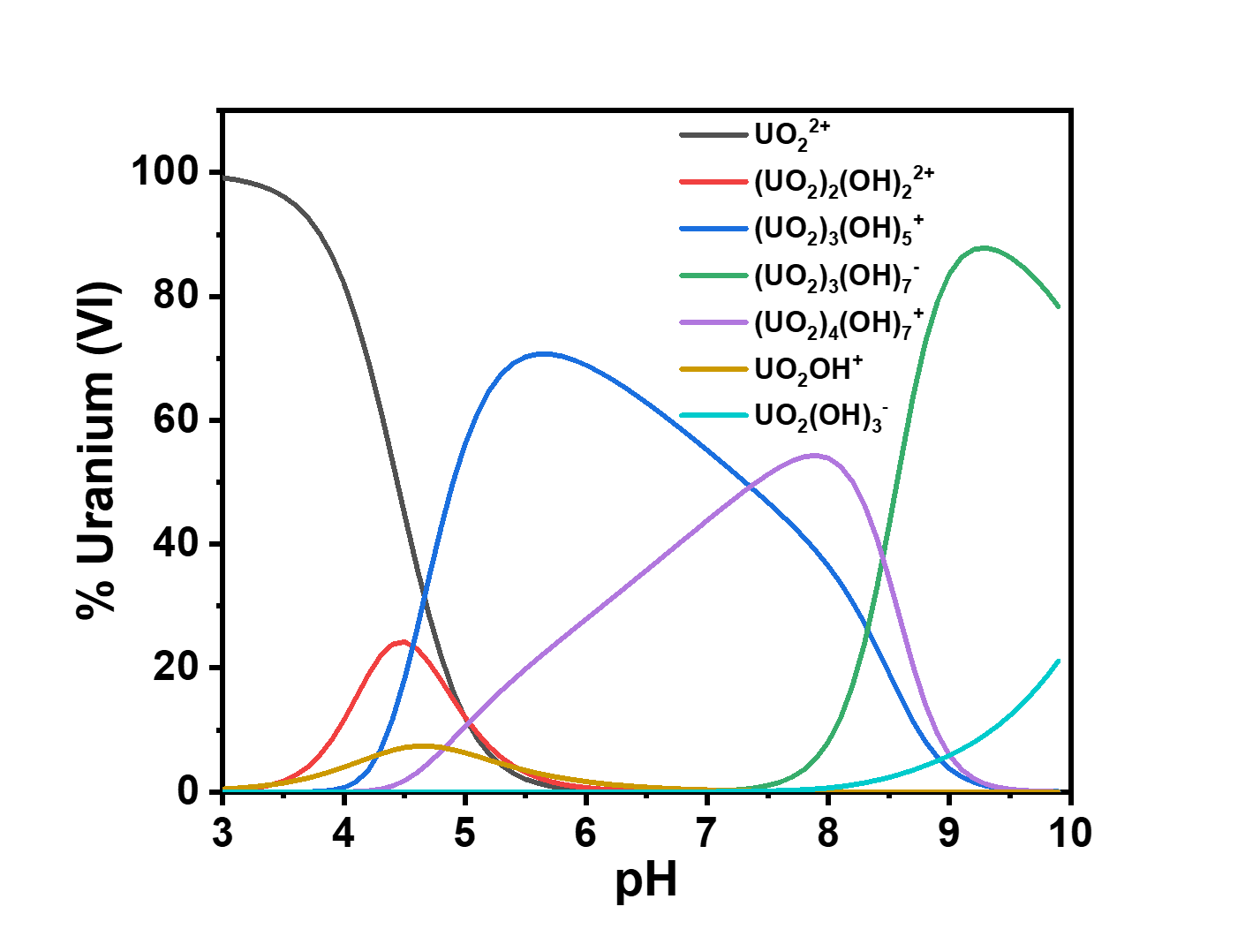
**

## Figure S26. The existence species of uranium ions under different pH conditions.

Figure S27. The influence of different pH conditions on the uranium extraction performance of electrodes. (a) The variation of uranium extraction rate of electrodes under different pH conditions over time; (b) Uranium extraction capacities of electrodes under different pH conditions within 5 hours.


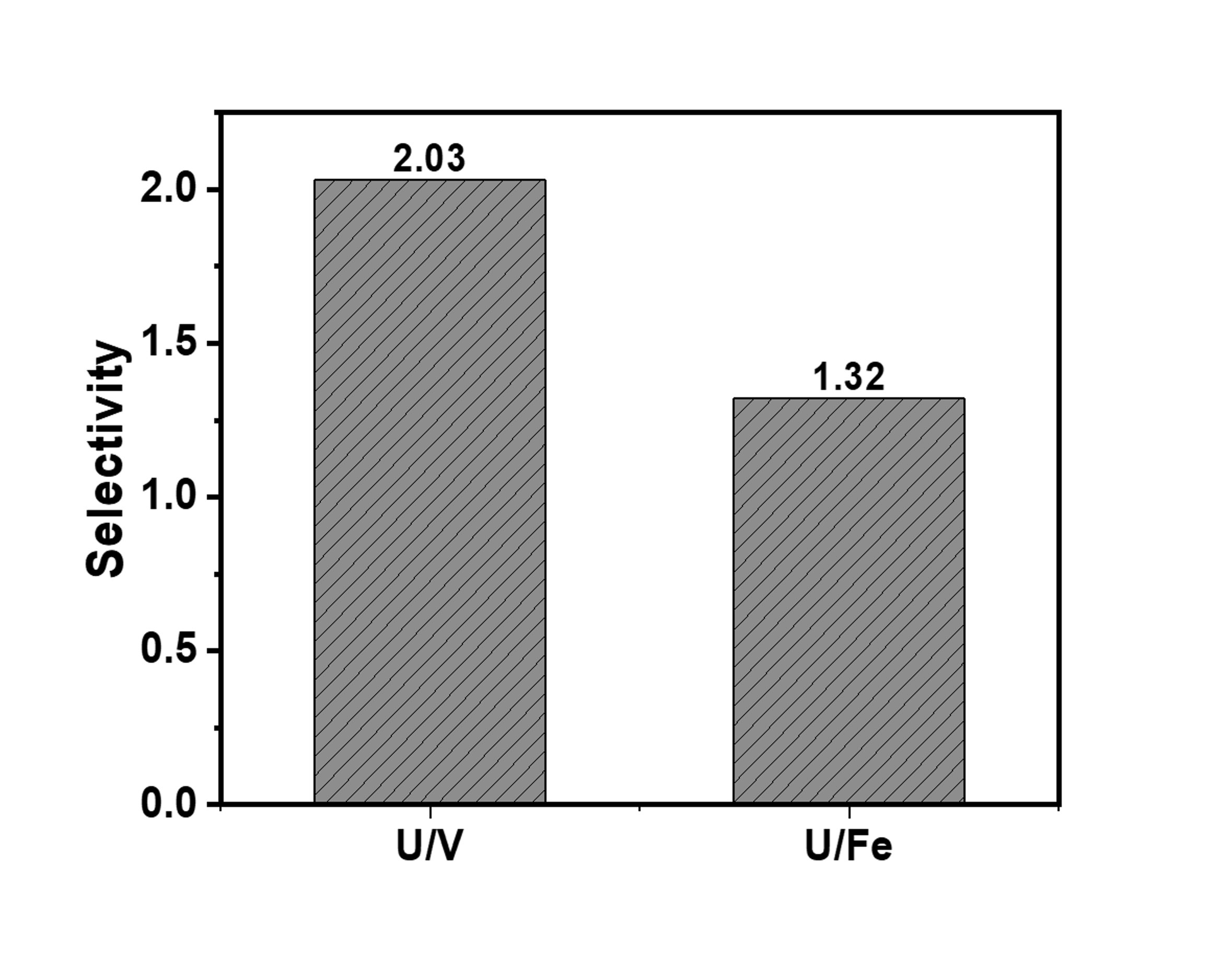


## Figure S28. Ion selectivity of AOPIM-CNT electrode.

## Figure S29. (a) CV curves of the AOPIM-CNT electrode in U-spiked and unspiked NaCl solutions; (b) CV curves of the electrode in U-spiked and unspiked natural seawater; (**c**) The variation of uranium extraction rate over time under different voltages.

## Figure S30. (a) XRD spectra of uranyl nitrate; (b) Raman spectra of uranyl nitrate at 532 nm wavelength; (c) Raman spectra of pristine AOPIM-CNT and electrode after electrochemical precipitation at 532 nm wavelength; (d) Quasi-in situ Raman spectra of electrode after 48 hours of uranium extraction under different electric field strengths; (e) XPS survey spectrum of AOPIM-CNT after the uranium extraction; (f) high-resolution U 4*f* spectra of AOPIM-CNT after the uranium extraction.

**
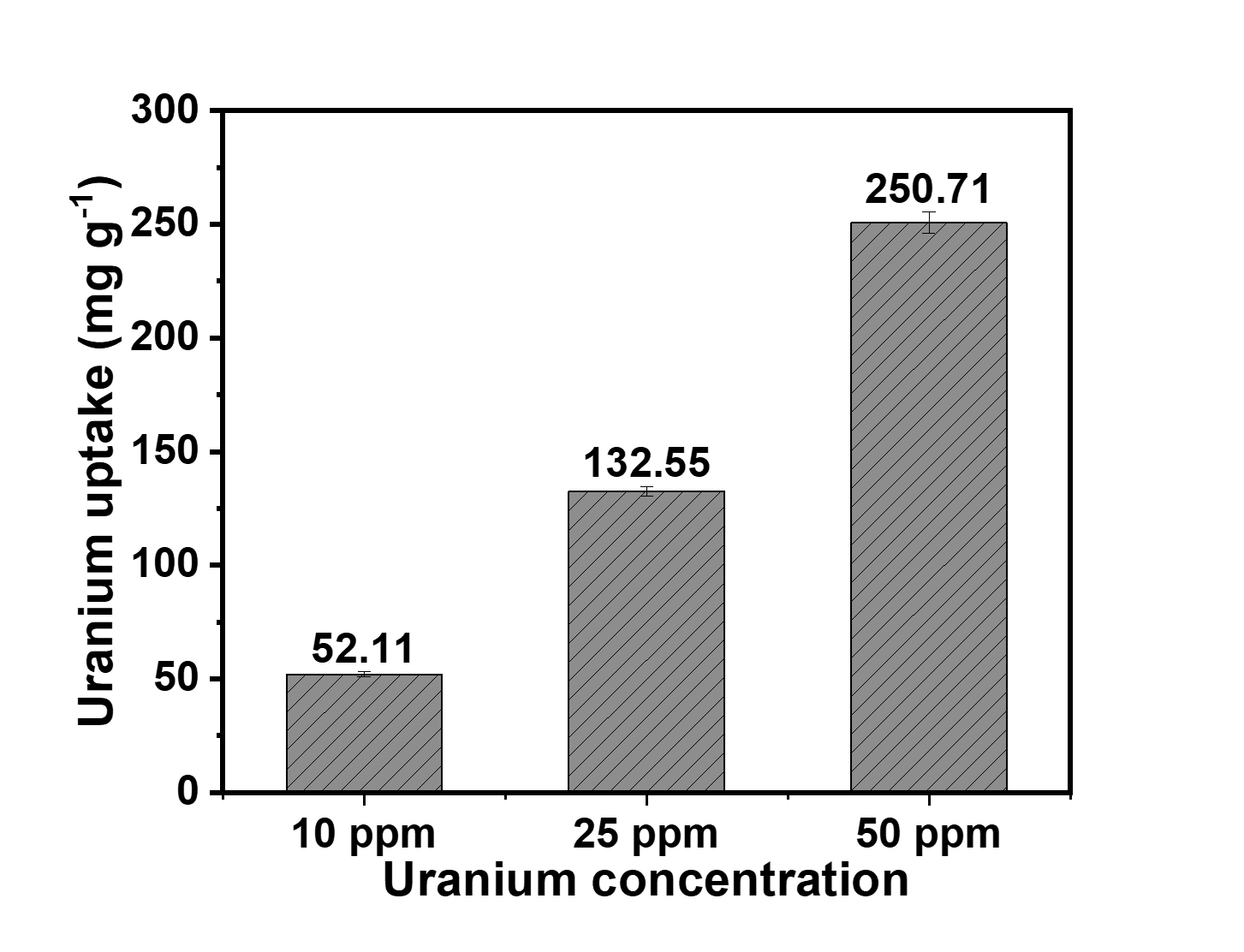
**

## Figure S31. Uranium extraction capacities in 24 hours under different concentrations of uranium-spiked seawater.

## Figure S32. AOPIM-CNT electrode uranium uptake and elution with NaHCO_3_ in (a) 50 ppm uranium-spiked fresh water and (b) seawater with varying uranium concentrations.


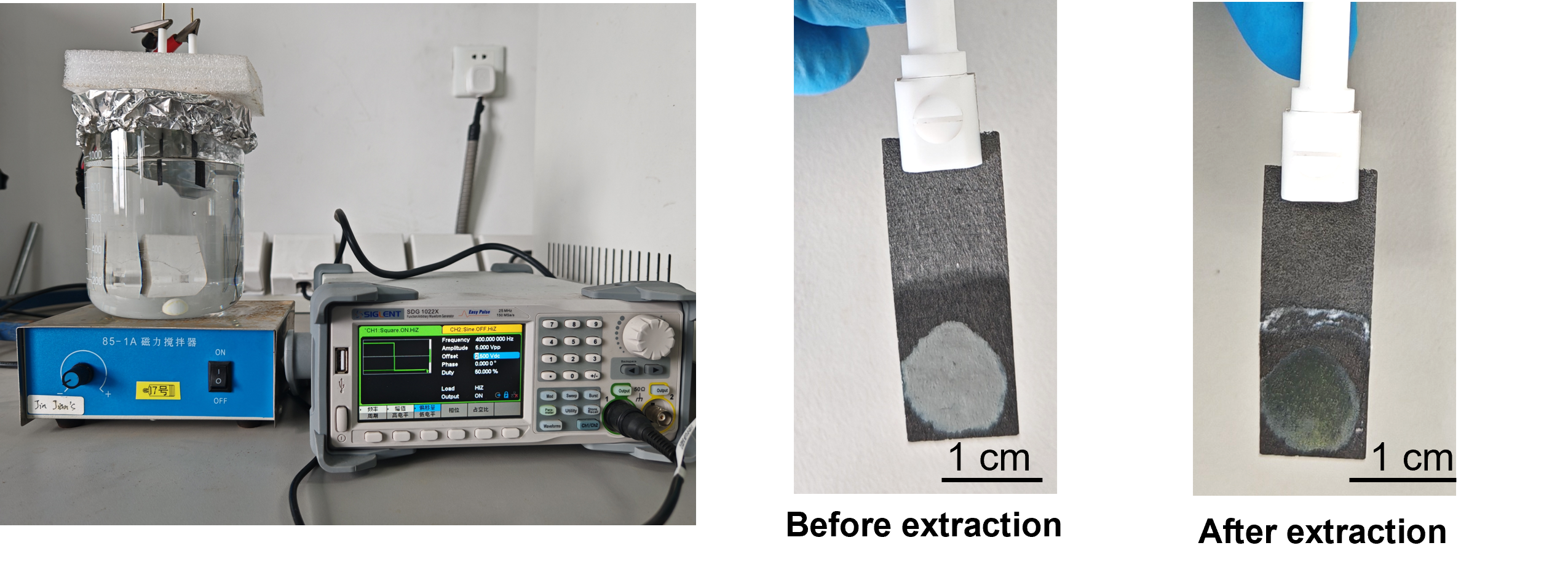


## Figure S33. Optical photos of real seawater uranium extraction equipment, and working electrode before and after extraction.

## Table S1. Formulation details of various AOPIM-CNT electrode slurry.

| CNTs content | NMP (g) | AOPIM-1 (g) | CNTs (g) |
| --- | --- | --- | --- |
| 0% CNTs | 9 | 1 | 0 |
| 2% CNTs | 9 | 1 | 0.02 |
| 5% CNTs | 9 | 1 | 0.05 |
| 10% CNTs | 9 | 1 | 0.1 |
| 20% CNTs | 9 | 1 | 0.2 |
| 30% CNTs | 9 | 1 | 0.3 |
| 40% CNTs | 9 | 1 | 0.4 |

## Table S2. Elemental concentration of U, V, and Fe in natural seawater and ion selectivity test feed in this study.

| Element | U | V | Fe |
| --- | --- | --- | --- |
| Concentration in seawater (ppb) | 3.3 | 1.5~2.5 | 1~2 |
| Concentration in test (ppm) | 3.3 | 2 | 1.5 |

## Table S3. Performance comparison with reported systems for uranium extraction from unspiked natural seawater.

| Materials | Strategy | Volume of seawater (L) | Time (day) | U uptake (mg g^-1^) | U uptake from 1 L sea water (μg g^-1^ day^-1^) | Reference |
| --- | --- | --- | --- | --- | --- | --- |
| Zn^2+^-PAO hydrogel | Adsorption | 1000 | 7 | 4.83 | 0.69 | 1 |
| H-ABP | Adsorption | - | 90 | 11.5 | - | 2 |
| AO-based nanotube | Adsorption | 100 | 30 | 9.01 | 3.01 | 3 |
| AOPIM | Adsorption | 100 | 28 | 9.03 | 3.23 | 4 |
| MISS-PAF-1 | Adsorption | - | 56 | 5.79 | - | 5 |
| Zn^2+^–PAO hydrogel | Adsorption | 1000 | 28 | 9.23 | 0.32 | 1 |
| AOP@PPLA | Adsorption | 50 | 35 | 10.31 | 5.89 | 6 |
| UIO-66-NH-AO | Adsorption | 25 | 8 | 5.2 | 26 | 7 |
| POP2-PO_3_H_2_ | Adsorption | 18.9 | 56 | 5.01 | 4.73 | 8 |
| PPH−OP | Adsorption | 100 | 21 | 7.63 | 3.63 | 9 |
| i-MZIF90@UIO-66 | Adsorption | 60 | 25 | 28.2 | 18.8 | 10 |
| PN-300 nanotubes | Adsorption | 25 | 15 | 7.01 | 18.69 | 11 |
| PAO@PAA-Na | Adsorption | 100 | 20 | 17.8 | 8.9 | 12 |
| PAO-Co | Adsorption | 100 | 56 | 9.7 | 1.73 | 13 |
| PE-PAO@ADH | Adsorption | 25 | 30 | 6.76 | 9.01 | 14 |
| BP-PAO | Photoinduce | 100 | 56 | 11.76 | 2.1 | 2 |
| VA-PG | Photocatalytic | 100 | 32 | 13.63 | 4.25 | 15 |
| GO-BPG-GLACS | Photothermal | 100 | 14 | 9.18 | 6.55 | 16 |
| 1-FeOOH/Ni@P | DC | 60 | 7 | 13.2 | 31.42 | 17 |
| B:Cu-PO_4_ | DC | 10 | 0.33 | 0.7 | 212.12 | 18 |
| MICOF-14 | DC | 100 | 5 | 20.8 | 41.6 | 19 |
| SBMA-MPAO | DC | 15 | 1 | 18.32 | 305 | 20 |
| Mo@PAF-6 | DC | 20 | 56 | 24.21 | 21.6 | 21 |
| CF@MTPN | DPST | 20 | 0.21 | 0.75 | 181 | 22 |
| MIGPAF-13 | AACE | 2 | 56 | 16.2 | 145 | 23 |
| TFPM-PDAN-AO | HW-ACE | 50 | 20 | 12.8 | 12.8 | 24 |
| PAO | HW-ACE | 4 | - | 1.5μg | - | 25 |
| In–Nx–C–R | HW-ACE | - | 2 | 12.7 | - | 26 |

(Continued Table S3)

| Materials | Strategy | Volume of seawater (L) | Time (day) | U uptake (mg g^-1^) | U uptake from 1 L sea water (μg g^-1^ day^-1^) | Reference |
| --- | --- | --- | --- | --- | --- | --- |
| Fe–Nx–C–R | HW-ACE | 2.4 | 1 | 1.2 | 500 | 27 |
| PAF-144-AO | HW-ACE | - | 24 | 12.6 |  | 28 |
| AO-PIM-CNT | HW-ACE | 1 | 1 | 1.09 | 1090 | This work |
| AO-PIM-CNT | HW-ACE | 5 | 5 | 5 | 200 | This work |

# References

1. B. Yan, C. Ma, J. Gao, Y. Yuan, N. Wang, *Adv. Mater.* **2020**, 32, e1906615.

2. X. Xu, H. Zhang, J. Ao, L. Xu, X. Liu, X. Guo, J. Li, L. Zhang, Q. Li, X. Zhao, B. Ye, D. Wang, F. Shen, H. Ma, *Energy Environ. Sci.* **2019**, 12, 1979-1988.

3. S. Zhao, Y. Yuan, Q. Yu, B. Niu, J. Liao, Z. Guo, N. Wang, *Angew. Chem. Int. Ed. Engl.* **2019**, 58, 14979-14985.

4. L. Yang, H. Xiao, Y. Qian, X. Zhao, X.-Y. Kong, P. Liu, W. Xin, L. Fu, L. Jiang, L. Wen, *Nat. Sustain.* **2021**, 5, 71-80.

5. Y. Yuan, Q. Meng, M. Faheem, Y. Yang, Z. Li, Z. Wang, D. Deng, F. Sun, H. He, Y. Huang, H. Sha, G. Zhu, *ACS Cent. Sci.* **2019**, 5, 1432-1439.

6. Z. Li, Z. Yu, Y. Wu, X. Wu, Y. Wan, Y. Yuan, N. Wang, *Chem. Eng. J.* **2020**, 390, 124648.

7. L. Ma, J. Gao, C. Huang, X. Xu, L. Xu, R. Ding, H. Bao, Z. Wang, G. Xu, Q. Li, P. Deng, H. Ma, *ACS Appl. Mater. Interfaces* **2021**, 13, 57831-57840.

8. Q. Sun, Y. Song, B. Aguila, A. S. Ivanov, V. S. Bryantsev, S. Ma, *Adv. Sci.* **2021**, 8, 2001573.

9. Y. Yuan, Q. Yu, M. Cao, L. Feng, S. Feng, T. Liu, T. Feng, B. Yan, Z. Guo, N. Wang, *Nat. Sustain.* **2021**, 4, 708-714.

10. S. Mollick, S. Saurabh, Y. D. More, S. Fajal, M. M. Shirolkar, W. Mandal, S. K. Ghosh, *Energy Environ. Sci.* **2022**, 15, 3462-3469.

11. L. Zhao, S. Wang, G. Wang, L. Cai, L. Sun, J. Qiu, *ACS Nano* **2024**, 18, 11804-11812.

12. Y. Huang, S. Zou, S. Lin, B. Na, Z. Li, S. Zhang, *J. Mater. Chem. A* **2024**, 12, 28390-28397.

13. W. Sun, L. Feng, J. Zhang, K. Lin, H. Wang, B. Yan, T. Feng, M. Cao, T. Liu, Y. Yuan, N. Wang, *Adv. Sci.* **2022**, 9, e2105008.

14. Y. Yao, J. Liao, X. Xu, C. Huang, M. Fu, K. Chen, L. Ma, J. Han, L. Xu, H. Ma, *J. Mater. Chem. A* **2024**, 12, 10528-10538.

15. T. Liu, R. Zhang, M. Chen, Y. Liu, Z. Xie, S. Tang, Y. Yuan, N. Wang, *Adv. Funct. Mater.* **2021**, 32, 2111049.

16. T. Li, X. Lin, Z. Zhang, L. Yang, Y. Qian, L. Fu, S. Zhou, W. Chen, Q. Wang, X. Li, X. Y. Kong, H. Xiao, L. Jiang, L. Wen, *Adv. Funct. Mater.* **2023**, 33, 2212819.

17. T. Li, Z. Yan, S. Chen, Y. Song, X. Lin, Z. Zhang, L. Yang, X. He, Y. Qian, S. Zhou, X. Li, Q. Wang, X.-Y. Kong, L. Jiang, L. Wen, *Adv. Funct. Mater.* **2025**, 35, 2412349.

18. J. Li, C. Jiao, Y. Lin, Y. Li, Z. Qian, H. Liu, T. Chen, Y. Liu, R. He, W. Zhu, *Appl. Catal. B Environ.* **2024**, 347, 123770.

19. C. Zhang, Z. Wang, R. Ma, J. Cao, X. Ruan, D. Cao, Y. Song, S. Chen, Y. Song, F. Wang, Y. Yuan, Y. Yang, G. Zhu, *Adv. Funct. Mater.* **2025**, 35, 2412712.

20. H. Li, X. Tang, Q. Xian, S. Zuo, Y. Zeng, C. Xu, J. Wen, Water Res. **2026**, 292, 125347.

21. J. Cao, W. Liu, C. Zhang, Y. Zheng, Y. Yang, D. Cao, Y. Song, W. Chen, L. Wen, X. Kong, H. Ma, S. Chen, Y. Song, F. Wang, Y. Yuan, Y. Wang, G. Zhu, Adv. Funct. Mater. **2025**, 36, e09223.

22. J. Peng, Q. Chen, Z. Zhu, J. He, Q. Fan, I. Shakir, Z. Jiang, X. Sun, J. Feng, J. Mater. Chem. A **2026**, 14, 11482-11492.

23. Z. Wang, R. Ma, Q. Meng, Y. Yang, X. Ma, X. Ruan, Y. Yuan, G. Zhu, *J. Am. Chem. Soc.* **2021**, 143, 14523-14529.

24. C.-R. Zhang, J.-X. Qi, W.-R. Cui, X.-J. Chen, X. Liu, S.-M. Yi, C.-P. Niu, R.-P. Liang, J.-D. Qiu, Sci. China Chem. 2022, 66, 562.

25. C. Liu, P.-C. Hsu, J. Xie, J. Zhao, T. Wu, H. Wang, W. Liu, J. Zhang, S. Chu, Y. Cui, *Nat. Energy* **2017**, 2, 17007.

26. X. Liu, Y. Xie, M. Hao, Z. Chen, H. Yang, G. I. N. Waterhouse, S. Ma, X. Wang, *Adv. Sci.* **2022**, 9, e2201735.

27. H. Yang, X. Liu, M. Hao, Y. Xie, X. Wang, H. Tian, G. I. N. Waterhouse, P. E. Kruger, S. G. Telfer, S. Ma, *Adv. Mater.* **2021**, 33, e2106621.

28. D. Chen, Y. Li, X. Zhao, M. Shi, X. Shi, R. Zhao, G. Zhu, *ACS Cent. Sci.* **2023**, 9, 2326-2332.
